# Fine-Mapping of a Wild Genomic Region Involved in Pod and Seed Size Reduction on Chromosome A07 in Peanut (*Arachis hypogaea* L.)

**DOI:** 10.3390/genes11121402

**Published:** 2020-11-25

**Authors:** Mounirou Hachim Alyr, Justine Pallu, Aissatou Sambou, Joel Romaric Nguepjop, Maguette Seye, Hodo-Abalo Tossim, Yvette Rachelle Djiboune, Djibril Sane, Jean-François Rami, Daniel Fonceka

**Affiliations:** 1 Centre d’Etude Régional pour l’Amélioration de l’Adaptation à la Sécheresse (CERAAS), Institut Sénégalais de Recherches Agricoles (ISRA), Route de Khombole, Thiès BP 3320, Senegal; alyr.hachim@gmail.com (M.H.A.); sambou.aissatou@yahoo.fr (A.S.); maxseye@gmail.com (M.S.); aristossim@yahoo.fr (H.-A.T.); rachyveta@yahoo.fr (Y.R.D.); 2 Département de Biologie Végétale, Faculté des Sciences et Techniques, Université Cheikh Anta Diop de Dakar, Dakar BP 5005, Senegal; djisane5@gmail.com; 3 CIRAD, UMR AGAP, F-34398 Montpellier, France; justinepallu42@gmail.com (J.P.); joel-romaric.nguepjop@cirad.fr (J.R.N.); rami@cirad.fr (J.-F.R.); 4 AGAP, Univ Montpellier, CIRAD, INRAE, Institut Agro, 34398 Montpellier, France; 5 CIRAD, UMR AGAP, Thies BP 3320, Senegal

**Keywords:** peanut, domestication, SSR, SNP, NILs, QTL fine-mapping, pod and seed size, candidate genes

## Abstract

Fruit and seed size are important yield component traits that have been selected during crop domestication. In previous studies, Advanced Backcross Quantitative Trait Loci (AB-QTL) and Chromosome Segment Substitution Line (CSSL) populations were developed in peanut by crossing the cultivated variety Fleur11 and a synthetic wild allotetraploid (*Arachis ipaensis* × *Arachis duranensis*)^4x^. In the AB-QTL population, a major QTL for pod and seed size was detected in a ~5 Mb interval in the proximal region of chromosome A07. In the CSSL population, the line 12CS_091, which carries the QTL region and that produces smaller pods and seeds than Fleur11, was identified. In this study, we used a two-step strategy to fine-map the seed size QTL region on chromosome A07. We developed new SSR and SNP markers, as well as near-isogenic lines (NILs) in the target QTL region. We first located the QTL in ~1 Mb region between two SSR markers, thanks to the genotyping of a large F_2_ population of 2172 individuals and a single marker analysis approach. We then used nine new SNP markers evenly distributed in the refined QTL region to genotype 490 F_3_ plants derived from 88 F_2_, and we selected 10 NILs. The phenotyping of the NILs and marker/trait association allowed us to narrowing down the QTL region to a 168.37 kb chromosome segment, between the SNPs Aradu_A07_1148327 and Aradu_A07_1316694. This region contains 22 predicted genes. Among these genes, *Aradu.DN3DB* and *Aradu.RLZ61*, which encode a transcriptional regulator STERILE APETALA-like (SAP) and an F-box SNEEZY (SNE), respectively, were of particular interest. The function of these genes in regulating the variation of fruit and seed size is discussed. This study will contribute to a better knowledge of genes that have been targeted during peanut domestication.

## 1. Introduction

The domestication of today’s food crops occurred approximately 10,000 years ago, with the beginning of agriculture. Human actions on wild crop relatives have drastically changed a wide range of morphological and physiological traits such as plant architecture, fruit size, seed dispersal, etc. These changes are collectively referred to as domestication syndrome [1,2]. Cultivated species generally have larger fruits or seeds compared to their wild ancestors, indicating that fruit and seed size are major agronomic traits that have been selected in crops during their domestication [2].

Peanut, one of the most economically important legumes in the world, is a recent allotetraploid (AABB) species, domesticated in South America. Cultivated peanut (*Arachis hypogaea*) resulted from a single hybridization event between the two wild diploid species *A. duranensis* (A genome) and *A. ipaensis* (B genome), followed by chromosomes doubling [3,4,5,6,7]. This polyploidization event first gave rise to the wild allotetraploid species *Arachis monticola* and, after subsequent domestication, to the cultivated species *A. hypogaea* [3,4,8,9]. Recent genome sequencing of all these species and their comparison confirm their phylogenetic relationships [5,6,7,9]. *A. duranensis*, *A. ipaensis*, *A. monticola* and the induced allotetraploid IpaDur1 (*A. ipaensis* KG30076 × *A. duranensis* V14167)^4x^ have similar seed size, and their seeds are smaller than the ones produced by the cultivated species, *A. hypogaea* [10]. This indicated that chromosome doubling is not directly responsible for increasing seed size in cultivated peanut although it has changed other traits such as plant architecture, biomass and photosynthetic pigments production during domestication [10,11]. Other genome modifications such as mutations, deletions, insertions and/or homeologous recombination could have been involved in the increase of pod size during peanut domestication [9]. Therefore, it is important to identify genes governing pod size variation and understand the genetic changes that occurred during the domestication of the peanut.

Interspecific populations are important genetic resources for mapping genomic regions involved in morphological changes that distinguish crops and their wild relatives. Fonceka et al. [12,13] have developed AB-QTL and CSSL populations from the cross between the cultivated variety Fleur11 and a wild synthetic allotetraploid that combines the genomes of *A. ipaensis* and *A. duranensis*. Using the AB-QTL population, these authors were able to detect three genomic regions on chromosomes A07, B02 and B05 where several QTLs for pod and seed size clustered. At these QTLs, the wild alleles explained 10% to 26% pod and seed size reduction. The authors hypothesized these regions as a target of human selection during peanut domestication. More recently, using the CSSL population, Tossim et al. [14] confirmed the QTL in the proximal region of chromosome A07 carried by the line 12CS_091 as involved in pod and seed size variation.

Several studies reported QTLs controlling pod/seed weight and size in peanut [12,15,16,17,18,19,20,21,22,23,24,25,26]. However, a few of them involved crosses between wild and cultivated species. Khera et al. [22] reported the mapping of QTLs in 2 interspecific AB-QTL populations involving (*A. ipaensis* × *A. duranensis*)^4x^ and (*A. kempff-mercadoi* × *A. hoehnei*)^4x^ as wild parents. These authors identified several regions involved in yield component traits, however, no seed size QTL was mapped in the cross involving (*A. ipaensis* × *A. duranensis*)^4x^.

QTL fine-mapping is one mean for identifying genes underlying phenotypic variation. It has been applied extensively in crop species for candidate genes identification and cloning [27]. It is a process by which the size of a QTL region (approximately 20 cM or more) is reduced to a few cM or less. The precision of fine-mapping approaches depend on the recombination frequency as well as the marker density in the QTL region [28,29]. Several marker and population types have been used in QTL fine-mapping, including biparental populations (RILs, NILs…), multiple founder populations (NAM, MAGIC…) and core-collections [29]. In peanut, Agarwal et al. [30] and Khan et al. [31] reported the fine mapping of QTLs involved in disease (ELS, LLS and rust) and *Aspergillus* resistances respectively, using RIL populations and high density SNP genetic maps. Luo et al. [20] succeeded in mapping major and stable QTLs related to weight and size of pods in 280 kb and 1.48 Mb intervals on chromosome A05 and A07, respectively, thanks to a genetic map constructed with 817 SSRs and a RIL population of 187 lines. Zhuang et al. [7] combined a bulk segregant analysis (BSA) and a RIL population to fine-map a QTL for seed size in 1 Mb interval on chromosome A07. As for other crop species, including rice [32,33,34], tomato [35] and *Medicago truncatula* [36], near-isogenic lines (NILs) have also been developed in peanut. They however have mostly been used to confirm QTLs involved in resistance to nematodes and rust [37,38,39]. At present, the genome sequences of several wild and cultivated peanut species are available [5,6,7], easing the development of thousands of molecular markers which, combined with resolutive mapping populations, can accelerate the fine mapping of QTLs.

In the present study, we developed a NIL population targeting a pod and seed size QTL region on chromosome A07. We used a two-step genotyping strategy combined with genotype/phenotype associations to increase the marker density and narrow down the QTL region to 168.37 kb interval containing 22 genes. Two interesting genes involved in the ubiquitin-proteasome pathway were highlighted and discussed. The discovery of the gene(s) involving in pod and seed size reduction will provide new information on peanut domestication and useful information for peanut geneticists and breeders.

## 2. Materials and Methods

### 2.1. Plant Materials

**Figure 1:**
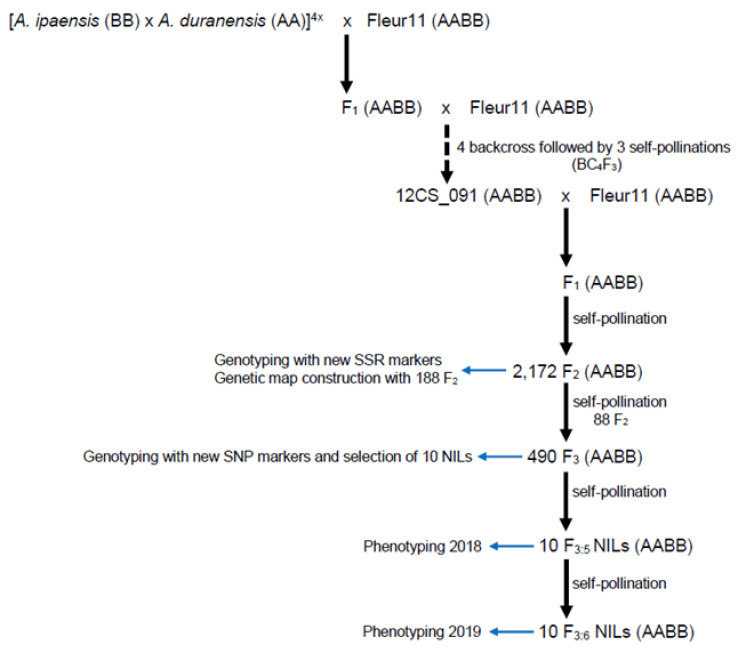
Selection scheme for NILs development.

The parents used in this study are the cultivated variety Fleur11 and the line 12CS_091. Fleur11 is an improved variety grown in Senegal. It is a Spanish type with an erect growth habit, low to moderate pod constriction, short cycle (90 days), high yielding, and moderate tolerance to drought [12,13,40]. The line 12CS_091 is a chromosome segment substitution line (CSSL) resulting from a backcross program involving Fleur11 and a synthetic wild allotetraploid (*A. ipaensis* KG30076 × *A. duranensis* V1416*7*)^4x^. Genotypically, this CSSL differs from Fleur11 by the introgression of a segment of *A. duranensis* chromosome in the proximal region of the chromosome A07 relative to the published genome sequence. Phenotypically, it has smaller seeds than Fleur11 [14]. From these parents, a large F_2_ population of 2172 individuals was developed by single cross followed by self-pollination of the F_1_ generation. 88 F_2_ plants were selected based on genotyping data and advanced to the F_3_ generation. A total of 490 F_3_ lines were used for SNP genotyping. Finally, 10 F_3_ plants were selected and advanced to produce NILs (F_3:5_ and F_3:6_) that were used for phenotyping and for identifying the candidate genes. Figure 1 shows the scheme used for developing the NILs.

### 2.2. DNA Isolation

DNA extraction was performed as described by Fonceka et al. [40]. Briefly, 20 mg of dried young leaves were ground 2 min in a Mixer Mill. The samples were then dissolved in 750 µL of MATAB buffer and incubated at 65 °C for 20 min in a water bath. A volume of 750 µL of chloroform-isoamyl alcohol (CIAA) was added to each sample, followed by centrifugation at 13,000 rpm for 20 min. A total of 600 µL of supernatant was harvested, transferred in a new tube and the DNA was precipitated by adding 600 µL of 2-propanol. After centrifugation, the pellets were washed with 500 µL of 70% ethanol and dried at ambient temperature before being dissolved in 500 µL of 1X Tris-EDTA. DNA extracted was stored at −20 °C for quantification and genotyping.

### 2.3. Development and Validation of the New SSR Markers

The QTL involved in pod and seed size reduction was located in the proximal region of chromosome A07 between RN13D04 and TC23E04 SSR markers [12,14]. This segment is approximately 5 Mb size and is tagged by four markers (RN13D04, Seq2E06, Seq5D05 and TC23E04) [12,40]. In a first step, we downloaded from PeanutBase (https://peanutbase.org/home) the entire sequence of *A. duranensis* located upstream of marker TC23E04 on chromosome A07. Then, all microsatellites of two, three or four nucleotides, and whose motifs were repeated at least 15 times, were searched using SSR Finder (http://fresnostate.edu/csm/faculty-research/ssrfinder/). Finally, primers were designed for 30 markers with the Primer3 [41]. SSR validation and polymorphism detection were assessed on 7 diploid species of A and B genomes (*A. batizocoi*, *A. duranensis*, *A. ipaensis*, *A. cardenasii*, *A. correntina*, *A. stenosperma* and *A. villosa*), four synthetic tetraploids ((*A. batizocoi* × *A. stenosperma*)^4x^, (*A. ipaensis* × *A. correntina)*^4x^, (*A. ipaensis* × *A. villosa*)^4x^, (*A. valida* × *A. duranensis*)^4x^), two cultivated varieties (Fleur11 and 73–33). Polymorphic SSRs between Fleur11 and *A. duranensis* were used for genotyping.

### 2.4. Development and Validation of New SNP Markers

Fifty new SNP markers were identified using GBS (genotyping by sequencing) data of the CSSL population developed by Fonceka et al. [13] and the “*Axiom-Arachis*” SNP array data [42]. KASP^®^ markers were developed for each SNP, using 50 bp flanking sequences and validated on *A. duranensis*, 12CS_091, Fleur11 and a subset of 21 F_3_ individuals of known genotype at selected SSR markers.

SNP genotyping was performed on a LightCycler480 (LC480). The amplification program was as follows: activation HotStart at 94 °C for 15 min, touchdown at 94 °C for 20 s then at 65 °C for 1 min (10× cycles, −0.8 °C/cycle), amplification at 94 °C for 20 s then at 57 °C for 1 min (28× cycles) and, finally, hold at 15 °C. Finally, fluorescence data of the 28th cycle were extracted from the LC480 and analysed with the snpclust package (https://github.com/jframi/snpclust).

### 2.5. Near-Isogenic Lines (NILs) Development

In a first step, the 2172 individuals of the F_2_ population were genotyped with 3 mapped SSRs (RN13D04, Seq2E06 and TC23E04), that tagged the wild chromosome segment containing the QTL of interest. 188 F_2_ that showed at least one recombination event between two adjacent markers were selected and genotyped with 23 polymorphic SSR markers developed in this study. All PCR amplifications were performed as described by Fonceka et al. [13]. The genotypic data were used for anchoring the new SSRs in the genetic map of the proximal region of chromosome A07, using Mapdisto software [43]. In a second step, 88 F_2_ out of 188 were selfed to produce 490 F_3_ that were genotyped with nine SNP markers developed in this study. SNP genotyping data were generated at CERAAS, using a LightCycler96 (Roche, Basel, Switzerland). Amplification and data analysis were performed using the same protocol as the one for SNP validation, except that the DNA and the mix volumes were doubled. Ten F_3_ individuals were identified as NILs based on the distribution of recombination events between RN13D04 and Seq2E06 markers as revealed by SNP genotyping data.

### 2.6. Phenotyping of F_2:3_ Families and the NILs

Hundred-seed weight (HSW) was first measured on selected F_2:3_ families in order to perform the single-marker analysis.

Ten NILs and the two parents (Fleur11 and 12CS_091) were evaluated under rainfed conditions, in Senegal, between July and October in 2018 and in 2019 at the ISRA-Nioro research station (13°45′28.8′′ N; 15°47′13.6′′ W). The experimental design was a randomized complete block design with three replications. In each replication, the NIL were arranged in rows of ten plants. The spacing was 30 cm between plants and 50 cm between rows. Weeds were managed manually before sowing and during all experiments. After harvesting and drying of pods, hundred-pod weight (HPW), hundred-seed weight (HSW), pod length (PL), pod width (PW), seed length (SL) and seed width (SW) were measured.

All statistical analyses of the phenotypic data were performed with R Core Team, Vienna, Austria (http://www.R-project.org/). The range, mean and standard deviation (SD) were calculated for each trait. An analysis of variance (ANOVA) was performed for each year to estimate the effects of genotypes and replications on each variable. From the results of the ANOVA, a Tukey multiple mean comparison test (HSD, for honestly significant difference) was applied to show differences between genotypes. All variables were also analyzed as a multi-environment trial (MET) using the following mixed model:

(1) $$y_{\mathrm{ijk}}\;=\;\mu \;+\;\alpha_{i}\;+\;\tau_{j}\;+\;{\left(\alpha \tau \right)}_{\mathrm{ij}}\;+\;\gamma_{\mathrm{jk}}\;+\;\varepsilon_{\mathrm{ijk}}$$

where *y_ijk_* is the response variable observed for the genotype *i* in the block *k* and the environment *j*; *μ* is the mean; *α_i_* is the effect of the genotype *i*; *τ_j_* is the effect of the year *j*; (*ατ*) is the interaction effect of the genotype *i* with the year *j*; *γ_jk_* is the effect of the block *k* within the year *j*; and *ε_ijk_* is the random error.

The genotype was treated as fixed effects while genotype by year as well as block effects were treated as random. Broad-sense heritabilities were then calculated for all traits using the following formula:

(2) $$h^{2}=\frac{\sigma_{G}^{2}}{\sigma_{G}^{2}+\sigma_{GY}^{2}+\sigma_{\varepsilon }^{2}}$$

where $\sigma_{G}^{2}$ is the genotypic variance, $\sigma_{GY}^{2}$ the genotype-by-year interaction variance and $\sigma_{\varepsilon }^{2}$ the residual variance.

### 2.7. Fine Mapping

A single-marker analysis was first performed using the SSR genotyping and HSW phenotyping data measured on selected F_2:3_ families. To this end, at each SSR locus, phenotypic data for lines homozygous for the cultivated Fleur11 or *A. duranensis* alleles were grouped, and then the phenotypic means for the two groups for the selected trait were compared using two-sample *t*-tests.

Phenotypic differences among NILs and between the NILs and their parents were checked vis-à-vis their genotypic constitution. Three groups of NILs were defined: similar phenotypic value to 12CS_091, intermediate phenotype, and similar phenotypic value to Fleur11. The QTL position was refined based on NILs phenotypic and genotypic differences and/or similarities. The most probable location of the QTL was identified and flanking SNP markers noted. MapChart [44] was used to draw the physical map of the markers and the position of the QTL of interest. Finally, sequence data between these two flanking SNP were download from the *A. duranensis* genome sequence available in PeanutBase (https://www.peanutbase.org/gbrowse_aradu1.0). All annotated genes were analysed in order to identify candidate genes.

### 2.8. Sequence Alignment of Selected Candidate Genes

Sequence alignment (coding DNA sequences (CDS) and proteins) was performed for two putative candidate genes using ClustalW (https://www.genome.jp/tools-bin/clustalw). The protein sequences corresponding to the genes were obtained using MEGA software 10.1.7 (https://www.megasoftware.net/). The gene sequences of *A. duranensis* were aligned against the homologous genes from *A. hypogaea* subs. *hypogaea* var. Tifrunner and *A. hypogaea* subs. *fastigiata* var. Shitouqi. Homologous genes were found by performing BLASTn and/or using the keyword search option in PeanutBase for var. Tifrunner (https://peanutbase.org) and in Peanut Genome Resource for var. Shitouqi (http://peanutgr.fafu.edu.cn/).

## 3. Results

### 3.1. Analysis of the Polymorphism of the New Genetic Markers

#### 3.1.1. New SSR Markers

We developed 30 new SSR markers in the target A07 chromosome region. Among these 30 SSRs, 28 were perfect repeats and 2 were composites. Twenty microsatellites had three nucleotide repeats and 10 were di-nucleotide repeats (Table S1). A total of 29 SSRs gave clear PCR amplification products. When considering all accessions used for the validation, 25 SSRs were polymorphic with an average number of 7.68 alleles per locus (Table S1). A total of 23 SSRs were polymorphic between Fleur11 and *A. duranensis*.

#### 3.1.2. New SNP Markers

Fifty new SNPs (31 from GBS data and 19 from the “*Axiom-Arachis*” SNP array) were developed in the target region. Among these SNPs, 23 were polymorphic, 23 were monomorphic and four did not amplify. Validation plots of some SNPs are shown in Figure S1 and a summary of the polymorphic SNP is presented in Table 1. GBS data provided more polymorphic markers than the *Axiom-Arachis* SNP array (67.7% vs. 10.5%). All markers developed from GBS data were co-dominant. Nine SNPs (Table 1) were used for the genotyping of the F_3_ plants.

### 3.2. Development of the NILs

In a first step, the genotyping of 2172 F_2_ with previously mapped SSRs markers allowed identifying 188 F_2_ that had at least one recombination between RN13D04 and Seq2E06 or between Seq2E06 and TC23E04. These F_2_ individuals were then genotyped with the 23 SSRs polymorphic between Fleur11 and *A. duranensis* (Table S1) of which 15 showed expected segregation profiles. All 15 new markers mapped on chromosome A07 between Seq2E06 and TC23E04 (Figure S2). We then performed a single marker analysis for HSW trait to identify the most likely marker interval that houses the QTL. The results from the two-sample *t*-tests showed that HSW reduction was significantly associated with *A. duranensis* alleles at RN13D04 (*p* = 0.0001) and at Seq2E06 markers (*p* = 0.01). No significant reduction of HSW was associated with *A. duranensis* alleles at the TC23E04 marker. These results indicated RN13D04-Seq2E06 interval (about 1 Mb) as the most likely location of the QTL.

**Figure 2:**
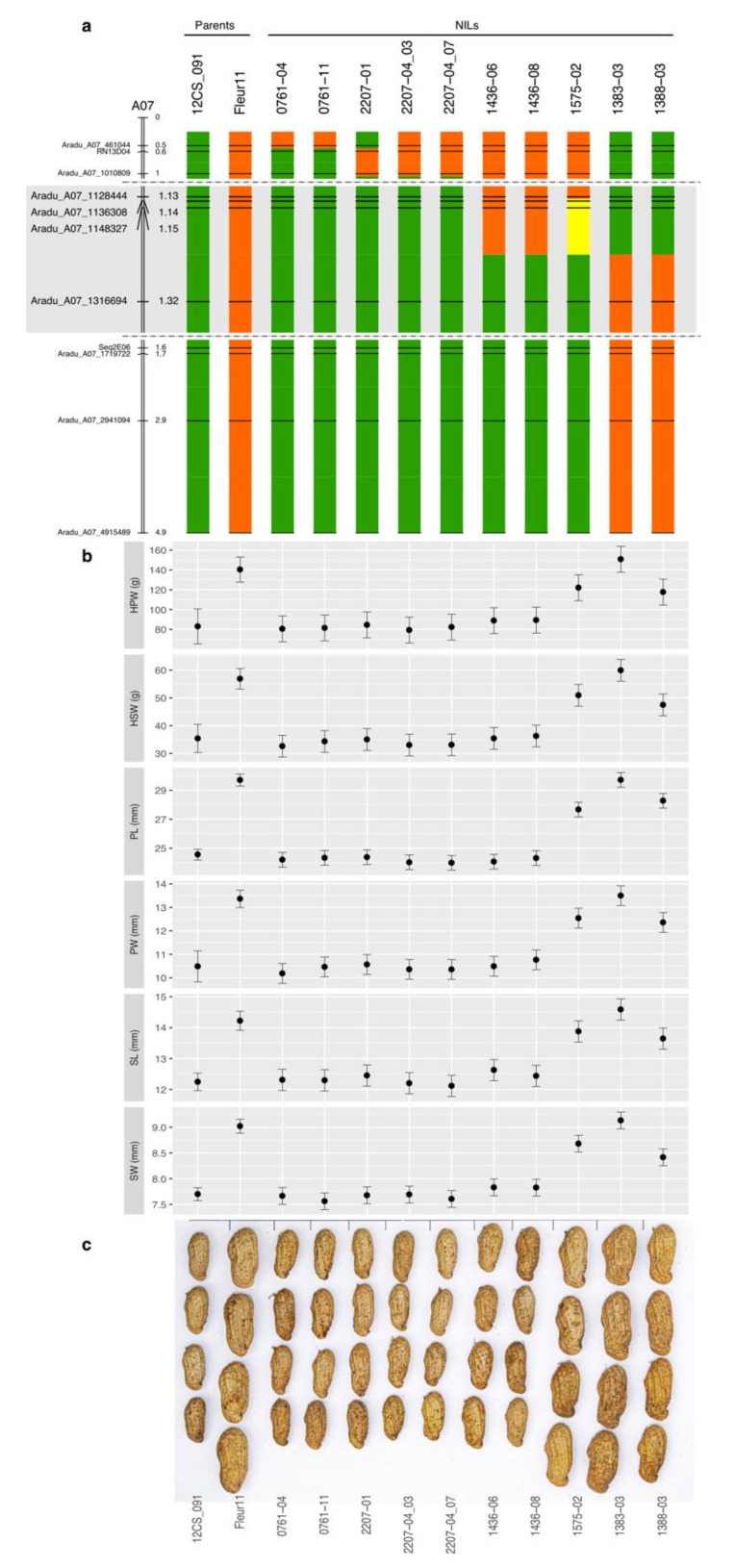
(**a**) Graphical genotypes of the NILs. The names and physical positions (in Mb) of the markers are shown on the left, and the names of NILs and parents at the top of the graph. Homozygous Fleur11 alleles are represented in orange and homozygous *A. duranensis* alleles in green. The region with an homeologous exchange between A and B genomes are represented in yellow. The two parents (Fleur11 and 12CS_091) are presented before NILs on the left. In order to reveal recombination points between very close markers, the vertical scale has been stretched in the region represented with a grey background. (**b**) Comparison of the mean values between the genotypes for the six measured traits. (**c**) Photography of harvested pods for the different NILs and their parents.

In a second step, we selected nine SNP markers based on their genomic position to cover the RN13D04-Seq2E06 region. One SNP was located upstream of RN13D04, 5 SNPs between RN13D04 and Seq2E06, and three SNPs downstream of Seq2E06. These SNPs and the SSR markers RN13D04 and Seq2E06 were used to genotype the 490 F_3_ derived from 88 selected F_2_. The genotypic data were used to identify F_3_ lines that showed at least one recombination event. Forty lines were identified out of which 10 were selected as NILs (Figure 2a). Among these 10 lines, nine were homozygous at all markers and one line (1575-02) had a particular genotypic constitution. In the plot of signal intensity generated for analysing the SNPs Aradu_A07_1136308 and Aradu_A07_1148327, this line was located between the cluster formed by the genotypes similar to Fleur11 and the cluster formed by heterozygous lines. To further investigate the genotype of the line 1575-02, we analysed the segregation patterns of the same two markers in 20 offspring derived from the self-fertilization of the line. Surprisingly, the segregation pattern of the progeny corresponded neither to that of a homozygous lines, nor to that of a heterozygous line, where the expected ratio would be ¼ homozygous for *A. duranensis* alleles, ½ heterozygous and ¼ for homozygous Fleur11 alleles. The offspring spread into two clusters; one with 15 individuals that clustered with Fleur11 and the other one with 5 individuals corresponding to the heterozygous genotypes. None of the offspring clustered with 12CS_091 and *A. duranensis* (Supplementary File S1, Figures A and B). The polymorphisms at SNPs Aradu_A07_1136308 and Aradu_A07_1148327 are C/A and A/G respectively. Based on the SNP clustering plots, Fleur11 is A^a^A^a^C^b^C^b^ at SNP Aradu_A07_1136308 and G^a^G^a^A^b^A^b^ at SNP Aradu_A07_1148327; the exponent letters a and b designed the peanut sub-genomes A and B, respectively. As shown in Supplementary File S1 (Figures A and B) the line 1575_02 was located in the same cluster than Fleur11. We, therefore, hypothesized that 1575_02 is derived from a homeologous recombination between the A and B genomes and is of C^a^A^a^C^b^A^a^ genotype at SNP Aradu_A07_1136308 and of A^a^G^a^A^b^G^a^ genotype at SNP Aradu_A07_1148327. The tetrasomic segregation ratio for SNP Aradu_A07_1136308 and the expected phenotype in the SNP plot of signal intensity is shown in Supplementary File S1, Tables B–F). This ratio fits well with a homeologous recombination with a gamete lethality or adverse selection model that can be found in segmental allopolyploids [45,46].

### 3.3. Phenotypic Variations of Pod- and Seed-Related Traits in NILs

The ten NILs were phenotyped in 2018 and in 2019 along with their parents. As expected, the small seed size donor parent 12CS_091 exhibited significantly smaller pod and seed sizes than Fleur 11 (Figure 2b,c and Figure S3). HPW, PL and PW ranged from 61.57 to 170.69 g, from 23.45 to 30.48 mm and from 9.51 to 14.09 mm, respectively. HSW, SL and SW ranged from 27.00 to 64.93 g, 11.69 to 15.04 mm and 7.43 to 9.32 mm, respectively. All measured traits had very high heritability (Table 2). The results of the single environment analysis and of the MET analysis showed a significant genotype effect for all traits (Table 2 and Table S2). Moreover, a significant genotype by year effect was found for HPW and HSW traits (Table 2). The comparison between lines and their parents showed that line 1383-03 produced pods and seeds as large as those of Fleur11 variety, and that the lines 0761-04, 0761-11, 1436-06, 1436-08, 2207-01, 2207-04_03 and 2207-04_07 produced pods and seeds statistically similar to those of 12CS_091 (Figure 2b and Figure S3). However, the two lines 1388-03 and 1575-02 had an intermediate phenotype for all the traits. We defined three groups of NILs based on the phenotypic data: small pod/seed group including lines 0761-04, 0761-11, 1436-06, 1436-08, 2207-01, 2207-04_03 and 2207-04_07, large pod/seed group including 1383-03, and intermediate pod/seed group with lines 1388-03 and 1575-02. We hypothesize that the three phenotypic groups correspond to three different genotypic classes at the locus of interest: large pods and seeds (homozygous for Fleur11 allele), small pods and seeds (homozygous for 12CS_091 allele) and intermediate phenotype (both alleles). This phenotypic distribution of the NILs suggested the action of a single gene or a few tightly linked genes.

### 3.4. Fine-Mapping of the QTL and Identification of Candidate Genes

**Figure 3:**
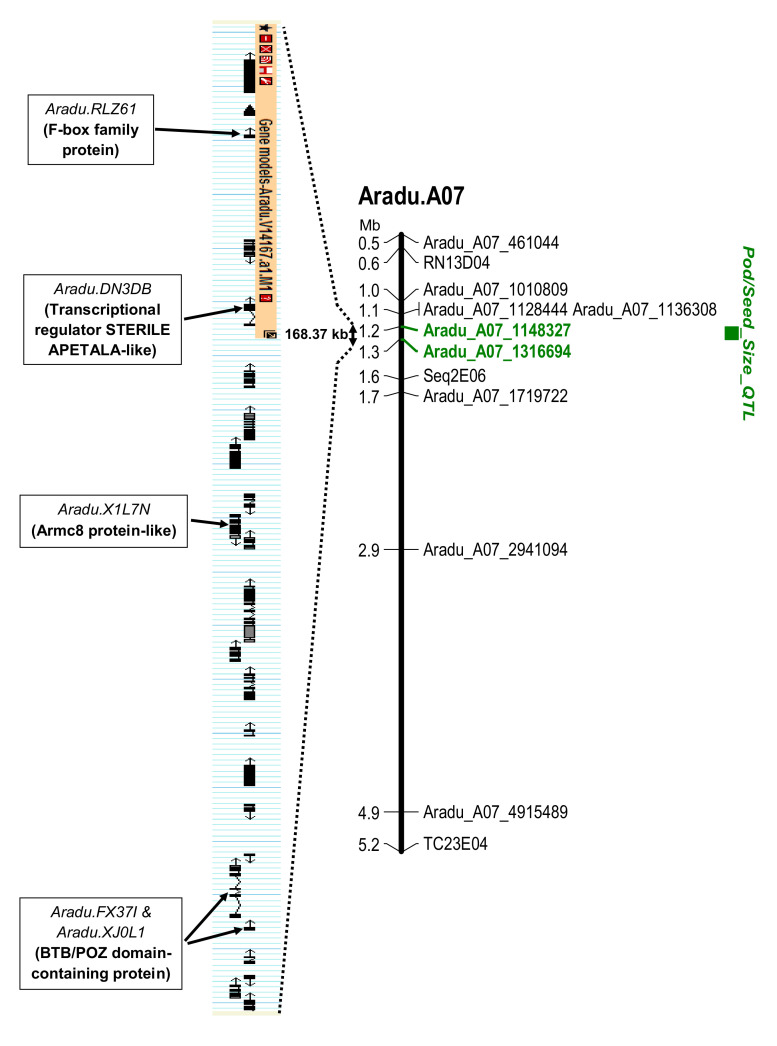
Position of the QTL responsible for reducing weight and size of pods and seeds on chromosome A07 and distribution of the candidate genes in the target region. The markers delimiting the QTL for pod/seed weight and size (*Pod/Seed Size_QTL*) reduction region are designated in green.

As shown in Figure 2a, the wild fragment responsible for pod and seed size reduction was subdivided into smaller fragments carried by different NILs. When combining the phenotyping and the genotyping results, we observed that the seven lines with small seeds (0761-04, 0761-11, 1436-06, 1436-08, 2207-01, 2207-04_03 and 2207-04_07) had introgressions of different size on chromosome A07 (Figure 2). However, although these NILs carried wild chromosome segments of different sizes, they all differed from the large seed size NIL 1383-03 by their genotypic constitution at the region located downstream of SNP Aradu_A07_1316694 (Figure 2). In this region, the seven NILs are homozygous for *A. duranensis* alleles while 1383-03 is homozygous for Fleur11 alleles. This finding allowed us excluding the chromosome region spanning from the top of the chromosome to SNP Aradu_A07_1148327 from the region that can carry the gene(s). The line 1388-03 appeared to have the same genotype than 1383-03, but had an intermediate phenotype for all the traits measured. These two lines could, however, differ in the location of the recombination event between SNP Aradu_A07_1148327 and Aradu_A07_1316694. Interestingly, the line 1575-02, which has a genotypic constitution similar to the small-seeded lines 1436-06 and 1436-08 but with a homeologous recombination in the region delimited by SNP Aradu_A07_1148327 and Aradu_A07_1316694, also had intermediate phenotypes for all the traits. These results suggest that the phenotypic differences between 1575-02 and 1436-06/08 on one hand and between 1383-03 and 1388-03, on the other hand, can be explained by different recombination event locations that occurred in the interval defined by the SNPs Aradu_A07_1148327 and Aradu_A07_1316694. Altogether, these results suggested, therefore, that the region containing the gene(s) involved in the pod and seed size reduction on chromosome A07, is localized in this interval. This interval is 168,367 nucleotides long based on *A. duranensis* chromosome A07 V14167 genome sequence (initial position A07: 1,148,327 and final position A07: 1,316,694) (Figure 3). This region contained 22 annotated and putative genes (https://www.peanutbase.org/gbrowse_aradu1.0). Among these genes, nine had no GO (Gene Ontology) term assigned and 13 were assigned at least to one GO term (Table 3). The molecular functions of the GO-assigned genes were related to protein binding (07), to catalytic activity (05) and to transmembrane transport and/or translation (01), while the biological processes in which they are participating are cellular and metabolic processes. Among these genes, four (04) were involved in the regulation of cell division and elongation through the ubiquitin-proteasome pathway. These genes are *Aradu.RLZ61*, *Aradu.DN3DB*, *Aradu.FX37I* (and/or *Aradu.XJ0L1*) and *Aradu.X1L7N*, which respectively encode an F-box family protein, a transcriptional regulator STERILE APETALA-like (SAP), a BTB/POZ domain-containing protein and an armadillo repeat-containing 8-like (armc8) protein (Figure 3 and Table 3).

### 3.5. Sequence Analysis of Selected Candidate Genes

**Figure 4:**
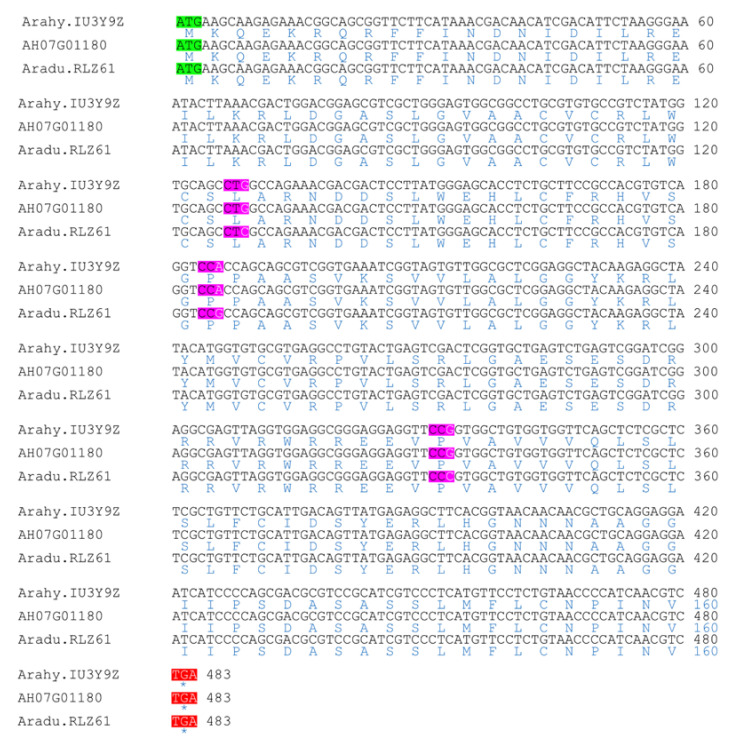
Sequence alignment of *A. duranensis SNE* (*Aradu.RLZ61*), *A. hypogaea* subs. *hypogaea* var. Tifrunner *SNE* (*Arahy.IU3Y9Z*) and *A. hypogaea* subs. *fastigiata* var shitouqi *SNE* (*AH07G01180*). The CDS sequences are figured in black and the corresponding protein sequences in blue. Three codons with SNP variation are highlighted in pink. The start codon is figured in green.

**Figure 5:**
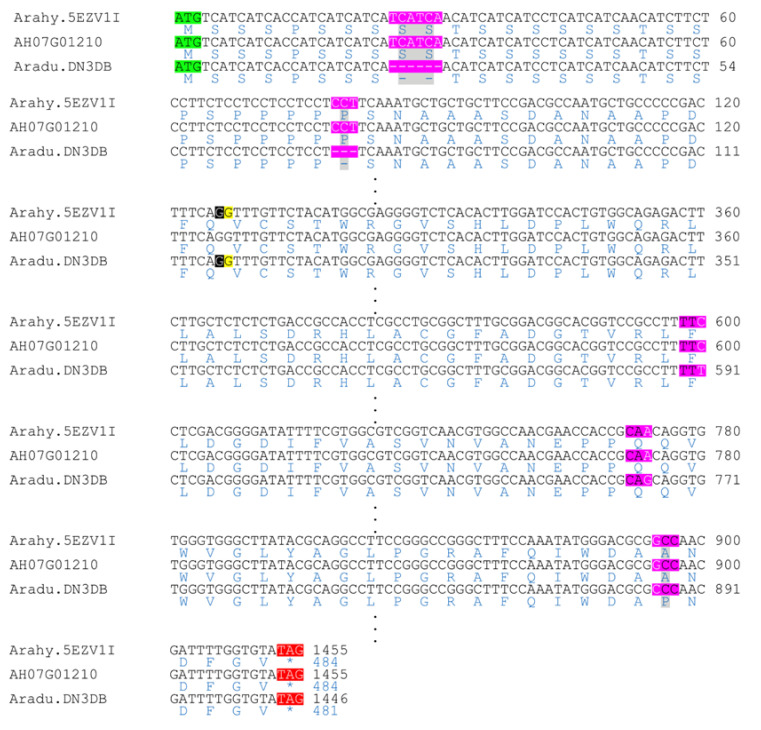
Sequence alignment of *A. duranensis SAP* (*Aradu.DN3DB*), *A. hypogaea* subs. *hypogaea* var. Tifrunner *SAP* (*Arahy.5EZV1I*) and *A. hypogaea* subs. *fastigiata* var Shitouqi *SAP* (*AH07G01210*). The position of the intron is delimited by the two nucleotides highlighted in black and yellow on each gene. The CDS sequences are figured in black and the corresponding protein sequences in blue. Deletions and SNP variation are highlighted in pink in the CDSs. In the protein sequences, the deletions and the substituted amino acid are highlighted in grey. The start codon is figured in green.

We compared the CDSs and corresponding protein sequences of two promising candidate genes in order to identify changes between the wild and cultivated species genes. *Aradu.RLZ61* has only one exon and is an ortholog of the *A. thaliana* SNEEZY (*At5g48170*) (Figure S4a). The alignment of the CDS sequences between *A. duranensis* SNEEZY (*Aradu.RLZ61*), subspecies *hypogaea* var. Tifrunner SNEEZY (*Arahy.IU3Y9Z*) and the subspecies *fastigiata* var. Shitouqi SNEEZY (*AH07G01180*) shows no difference between the subspecies and three SNP variations between the wild and cultivated species genes (Figure 4). These mutations are however synonymous because no change is observed in the protein sequences. The SNEEZY protein from *A. duranensis* and those of the two subspecies are all 160 amino acids long. We also analyzed *Aradu.DN3DB* gene. This gene encode a transcriptional regulator STERILE APETALA-like or SAP (Table 3 and Figure 3) and it is an orthologue of *M. truncatula* SLB1 (Figure S4b). Alignment of *Aradu.DN3DB* (*A. duranensis_SAP*), *Arahy.5EZV1I* (*A. hypogaea* subs. *hypogaea* var. Tifrunner *SAP*) and *AH07G01210* (*A. hypogaea* subs. *fastigiata* var. Shitouqi *SAP*) showed that there is no difference between the two subspecies sequences. However, a deletion of three codons in the *A. duranensis* CDS sequence, two at position 25–30 and one at 61–63, is observed (Figure 5). These codons code for two serines and one proline according to the protein sequences of the two cultivated subspecies of *A. hypogaea*. Moreover, three SNP variations, at positions 600, 774 and 895, on the *A. hypogaea* sequences were also observed. Two of these SNPs (positions 600 and 774) are synonymous. However, the citosine (C) to guanine (G) change at position 895 on the *A. hypogaea* sequences led to the substitution of proline (CCC) in *A. duranensis* to alanine (GCC) in *A. hypogaea*. *Aradu.DN3DB* and *Arahy.5EZV1I* consist of two exons separated by a long intron of 2527 bp and 2511 bp respectively. *Arahy.5EZV1I* and *AH07G01210* encode proteins of 484 amino acids, while *Aradu.DN3DB* encode a protein of 481 amino acids (Figure 5).

## 4. Discussion

### 4.1. NILs are Resolutive Genetic Material for Fine Mapping Applications

In our study, we developed a NIL population as a tool for fine mapping a pod/seed size QTL region on chromosome A07. NIL is a widely used genetic mapping population for QTL validation and for fine-mapping of QTL. Success of NILs in the fine-mapping approach depends essentially on the marker density and the frequency of recombinations in the QTL region [28]. We used a cost-effective two-step strategy for increasing marker density, checking for recombination events and for developing NILs. In the first step, previously mapped SSR markers were used to select a subset of 188 F_2_ plants that showed at least one recombination between two adjacent markers from a total of 2172 F_2_. This subset then was genotyped with 23 polymorphic newly developed SSRs. This allowed reducing by at least 86% the total number of data points that would have been generated if we had genotyped the entire F_2_ population with all SSR markers. In a second step, we used nine SNP markers located in a refined QTL region to genotype 490 F_3_ plants derived from 88 F_2_. The use of F_3_-derived heterozygous F_2_ plants allowed increasing the number of recombinations in the target region and selecting for relevant NILs. Finally, the NIL genotype/phenotype association allowed identifying a region of 168.37 kb as the most likely location of the gene(s). This interval is larger than what reported in other studies that used NIL to fine map QTL regions. For example, in rice, using backcross-derived NILs, Fan et al. [32] were able to delimit a QTL for grain weight and size in a segment of 7.9 kb using 1384 BC_3_F_2_ and 11 molecular markers. Similarly, Liu et al. [34] delimited a QTL for stigma exsertion rate in rice in an interval of 28.4 kb, using 3192 BC_4_F_2_ and eight molecular markers. In *M. truncatula*, four HIF (heterogeneous inbred families)-derived NILs and SSR markers allowed to fine mapping a QTL involved in the resistance of root disease in a fragment of 135 kb [36]. However, larger regions were also reported and it was not a major bottleneck for candidate gene identification. For example, Xue et al. [47] succeeded in cloning *Ghd7* gene in rice while the genomic region was narrowed to a large segment of 2284 kb.

In our study, we found that two NILs (1575-02 and 1388-03) had an intermediate phenotype across the two years of field evaluation. The segregation ratio of SNPs Aradu_A07_1136308 and Aradu_A07_1148327 in the offspring of line 1575-02 indicated a homeologous recombination between the A and B chromosomes, changing the genomes composition in the genomic region between the two SNPs from AsAsBcBc to AsAcBcAc (the A and B represent the peanut subgenomes and the s and c to the wild and cultivated origin, respectively). The change in genome composition could explain the alteration of the phenotype of line 1575-02 towards intermediary pod and seed size. Bertioli et al. [6] reported that homeologous exchanges are involved in modification of flowers colour in peanut. When considering the line 1388-03, it was genotypically identical to the line 1383-03 at all markers. They both presented one recombination between SNPs Aradu_A07_1148327 and Aradu_A07_1316694. However, in these lines, the exact location of the recombination events is unknown. In the hypothesis of the presence of two candidate genes in the region, the recombination event in line 1388-03 could have occurred between them, gathering in the same genotype an *A. duranensis* allele at one gene and a Fleur11 allele at the other. Assuming that the two genes acted additively, this could explain the intermediary phenotype observed in 1388-03 line. A graphical representation of the 2 genes model with additive action is shown in Supplementary Figure S5. This results reinforce the usefulness of NILs for deciphering the genetic components of complex phenotype [48].

### 4.2. Candidate Genes Associated with Seed Size are Found in the QTL Region

Few studies have reported QTLs for pod and seed size in the proximal region of the chromosome A07 in peanut. This region has first been reported housing a major QTL for pod and seed size-related traits by Fonceka et al. [12]. Luo et al. [20] also identified a similar region on chromosome A07 (from 0.06 to 1.54 Mb), as containing major, stable and co-localized QTLs involved in pod weight and size variation that explained up to 43.62% of phenotypic variation. This region colocalized with the pod and seed size QTL cluster reported by Chavarro et al. [26] around 0.63–1.03 Mb. Finally, Zhuang et al. [7] reported the same region on chromosome A07 (0.87 to 1.9 Mb) as containing a QTL that controlled pod and seed size. These authors reported a total of 99 genes among which 19 were putative candidates. In our study, a region of 168.37 kb size, delimited by two SNPs, starting from 1,148,327 bp to 1,316,694 bp, was identified on the chromosome A07 (Figure 3). Therefore, it is likely that this region contains major determinants of pod and seed size variation in peanut. In *A. duranensis* genome sequence, the region we identified contains 22 putative genes. Among the genes, four genes (i.e., *Aradu.RLZ61*, *Aradu.DN3DB*, *Aradu.FX37I* and *Aradu.X1L7N*) involved in the ubiquitin-proteasome mediated cell proliferation pathway were of particular interest. This pathway has been shown to regulate seed size in plants [49,50]. The genes, *Aradu.FX37I* (and/or *Aradu.XJ0L1*) and *Aradu.X1L7N*, encode a BTB/POZ domain-containing protein and an armadillo repeat-containing protein 8-like (armc8) respectively. The BTB/POZ domain-containing proteins have been described as specific SKP1/Cullin/F-box E3 ubiquitin ligase complex substrate adapters and armc8 is part of the CTHL complex protein (conserved C-terminal to LisH motif) which is involved in the regulation of microtubule dynamics and cell division [51,52,53]. To our knowledge, there is no evidence in the literature that these genes are associated with fruit or seed size variation. The gene *Aradu.RLZ61* encodes an F-box family protein similar to the F-box SNE (SNEEZY) protein. In *Arabidopsis*, the F-box SNE protein is involved in the regulation of gibberellin (GA) signalling. Its overexpression is associated with the loss of dwarf character in a mutant in which the gene *SLY1* (homolog of *SNE*) is not functional [54,55]. It was also shown that overexpression of *SNE* caused a decrease in DELLA RGA and GAI proteins [56]. These DELLA proteins were found to be differentially expressed during fruit development in *Arabidopsis* [56]. Studies of GA action are mostly limited to seed germination and plant growth although it has been hypothesized that it could be among the phytohormones that could, combined with auxin and ABA, control the final seed size in the *Medicago* model plant [57]. The role of GA in cell division and elongation has been established [58]. Thus, although the protein sequences of this gene are conserved between *A. duranensis* and *A. hypogaea* (Figure 4), the gene *Aradu.RLZ61* could possibly play a role in the pods and seeds size variation in peanut. Another interesting candidate is the gene *Aradu.DN3DB* which encodes a transcriptional regulator STERILE APETALA-like (SAP) protein. *SAP* gene was first identified in *Arabidopsis thaliana* as a flower regulator. The loss of function of this gene in a mutant causes severe aberrations in inflorescence, flower and ovule development, leading to sterile flowers with small petals [59]. Decrease of SAP activity in *Capsella rubella* shortens the period of cell proliferation and reduces the number of petal cells [60]. SAP was described as an F-box family protein and one of the components of a SKP1/Cullin/F-box E3 ubiquitin ligase complex, which controls not only flower size but also leaf and fruit size in *Arabidopsis*, by targeting the repressor factors PPDs (PEAPOD1 and PEAPOD2) and KIXs (kinase-inducible domain interacting proteins KIX8 and KIX9) for degradation [61,62]. Recently, in cucumber, *LITTLELEAF* an ortholog of *A. thaliana* SAP (*At*SAP), was associated with the variation of leaf, fruit, seed size and number of lateral branches by regulating mainly cell number [63]. In legumes, *BIG SEED1* (*BS1*) was described as homologue of PEAPOD1 and PEAPOD2. In *M. truncatula* and soybean, *BS1* suppression leads to an increase in leaf, fruit and seed size [64]. More recently, an ortholog of the *At*SAP (i.e., *SLB1*) was identified in *M. truncatula* and it has been shown that SLB1 interacts with BS1 and targets it for degradation, leading to an increase of leaves, fruits and seeds size [65]. SAP was reported to be over expressed in the embryo of a large-seeded *A. hypogaea* genotype compare to one that gave smaller seed [7]. Sequence alignment between *A. duranensis SAP* and those of the two cultivated subspecies (var. Tifrunner and var. Shitouqi) (Figure 5) showed that there are deletions of some codons in the serine/glycine rich region in the *A. duranensis* sequence. In addition, variations of three SNPs were also observed in the WD40 repeat-containing domain, two of which have no effect on the protein sequences. However, the other SNP at position 895 on the *A. hypogaea* sequence led to the substitution of a proline (CCC) in *A. duranensis* for alanine (GCC) in *A. hypogaea* (Figure 5). In SAP protein, the serine/glycine rich region is a target for phosphorylation and the WD40 repeat-containing domain has been suggested to coordinate protein-protein interactions [60,62]. Finally, sequence alignment of *A. hypogaea* SAP and *M. truncatula* SLB1 shows that these genes are orthologs (Figure S4) and the CDS is well conserved between the *A. hypogaea* subspecies we compared in this study. Taken together, the results presented in this study suggest that the transcriptional regulator STERILE APETALA-like is a good candidate gene involved in pod and seed size variation during peanut evolution. However, this gene needs to be validated for confirmation.

## 5. Conclusions

In the present study we increased the markers density in the proximal region of chromosome A07 by developing and mapping new SSR and SNP markers. The molecular markers and marker/traits association allowed to identifying different NILs and to narrowing down the QTL region involved in pod/seed size variation to a 168.37 kb segment containing 22 genes. Putative candidate genes were identified and discussed, among them, two were of particular interest. However, more studies are needed for their validation and for identifying with which other genes they interact. Discovery of the gene(s), involving in pod/seed size variation, will contribute to a better knowledge on genes targeted during the domestication of peanut and it will facilitate decisions in future peanut breading programs.

## Figures and Tables

**Table 1:** Polymorphic SNPs developed in this study.

Sources	Names	SNP	Positions on Aradu.A07 (bp)	Types
GBS data	Aradu_A07_213561	[G/T]	213511 to 213611	Co-dominant
	* Aradu_A07_461044	[C/G]	460994 to 461094	Co-dominant
	Aradu_A07_964804	[G/C]	964754 to 964854	Co-dominant
	* Aradu_A07_1010809	[G/A]	1010759 to 1010859	Co-dominant
	* Aradu_A07_1128444	[C/T]	1128394 to 1128494	Co-dominant
	* Aradu_A07_1136308	[C/A]	1136258 to 1136358	Co-dominant
	* Aradu_A07_1148327	[A/G]	1148277 to 1148377	Co-dominant
	* Aradu_A07_1316694	[C/T]	1316644 to 1316744	Co-dominant
	* Aradu_A07_1719722	[A/G]	1719672 to 1719772	Co-dominant
	Aradu_A07_2243243	[C/T]	2243193 to 2243293	Co-dominant
	Aradu_A07_2316522	[A/G]	2316472 to 2316572	Co-dominant
	Aradu_A07_2390033	[G/A]	2389983 to 2390083	Co-dominant
	Aradu_A07_2557764	[C/T]	2557714 to 2557814	Co-dominant
	* Aradu_A07_2941094	[G/A]	2941044 to 2941144	Co-dominant
	Aradu_A07_3132003	[T/C]	3131953 to 3132053	Co-dominant
	Aradu_A07_3705362	[G/A]	3705312 to 3705412	Co-dominant
	Aradu_A07_4010442	[A/G]	4010392 to 4010492	Co-dominant
	Aradu_A07_4215704	[G/T]	4215654 to 4215754	Co-dominant
	Aradu_A07_4506062	[G/A]	4506012 to 4506112	Co-dominant
	Aradu_A07_4621636	[C/T]	4621586 to 4621686	Co-dominant
	* Aradu_A07_4915489	[T/C]	4915439 to 4915539	Co-dominant
“*Axiom-Arachis*” SNP array	AX_147226901	[A/G]	1086149 to 1086249	Co-dominant
	AX_147226999	[C/T]	1854207 to 1854307	Dominant

* indicates the SNPs used to genotype the F_3_ plants.

**Table 2:** Descriptive statistics NILs evaluation in Nioro in 2018 and 2019 and ANOVA table of multi-environment mixed-model analysis.

	Nioro2018		Nioro2019		MET Analysis			
Traits	Range	Mean ± SD	Range	Mean ± SD	GEN F	GEN Pr (>F)	GEN:ENV Pr (>Chisq)	h^2^
**HPW (g)**	61.57–137.18	93.50 ± 22.34	79.36–170.69	109.89 ± 30.55	28.82	1.11 × 10^−6^ ***	6.68 × 10^−6^ ***	0.93
**PL (mm)**	23.60–30.48	26.10 ± 2.39	23.45–30.25	25.83 ± 2.44	155.18	3.61 × 10^−43^ ***	1.00 × 10^0^	0.99
**PW (mm)**	9.51–13.65	11.07 ± 1.42	10.02–14.09	11.68 ± 1.23	65.28	2.46 × 10^−9^ ***	3.84 × 10^−1^	0.98
**HSW (g)**	27.00–56.01	39.28 ± 9.26	32.34–64.93	43.93 ± 11.42	48.44	2.73 × 10^−8^ ***	5.23 × 10^−3^ **	0.97
**SL (mm)**	11.69–15.04	13.06 ± 0.87	11.78–14.73	12.87 ± 0.99	48.84	3.24 × 10^−9^ ***	1.64 × 10^−1^	0.98
**SW (mm)**	7.50–9.22	8.15 ± 0.58	7.43–9.32	8.06 ± 0.63	92.29	9.47 × 10^−12^ ***	8.60 × 10^−1^	0.99

HPW: hundred-pod weight, PL: pod length, PW: pod width, HSW: hundred-seed weight, SL: seed length, SW: seed width, SD: standard deviation. MET: multi-environment trial, GEN F: F Value of the genotype fixed effect, GEN Pr (>F): *p*-value associated to the F test of genotype fixed effect, GEN:YR Pr (>Chisq): *p*-value associated to likelihood ratio test of genotype by year random effect, h^2^: broad-sense heritability, ** and ***: significant at *p* < 0.005 and *p* < 0.001 respectively.

**Table 3:** Candidate genes found in the identified interval.

Gene Names	Positions on Aradu.A07 (bp)	Descriptions
*Aradu.WGI5L*	1144973 to 1150960	ATP binding; GTP binding; nucleotide binding; nucleoside-triphosphatases; IPR000767 (Disease resistance protein), IPR027417 (P-loop containing nucleoside triphosphate hydrolase); GO:0000166 (nucleotide binding), GO:0006952 (defence response), GO:0017111 (nucleoside-triphosphatase activity), GO:0043531 (ADP binding)
*Aradu.870BG*	1153343 to 1155460	DnaJ homolog subfamily B member 14-like [Glycine max]; IPR001623 (DnaJ domain), IPR024593 (Domain of unknown function DUF3444)
*Aradu.RLZ61*	1159085 to 1159567	F-box family protein; IPR001810 (F-box domain); GO:0005515 (protein binding)
*Aradu.SFU0J*	1178336 to 1181460	Phytochromobilin:ferredoxin oxidoreductase, chloroplastic-like isoform X2 [Glycine max]; IPR009249 (Ferredoxin-dependent bilin reductase); GO:0010024 (phytochromobilin biosynthetic process), GO:0050897 (cobalt ion binding), GO:0055114 (oxidation-reduction process)
*Aradu.DN3DB*	1190431 to 1194331	Transcriptional regulator STERILE APETALA-like [Glycine max]; IPR015943 (WD40/YVTN repeat-like-containing domain); GO:0005515 (protein binding)
*Aradu.BD60N*	1202798 to 1205874	Glucose-1-phosphate adenylyltransferase family protein; IPR011831 (Glucose-1-phosphate adenylyltransferase); GO:0005978 (glycogen biosynthetic process), GO:0008878 (glucose-1-phosphate adenylyltransferase activity), GO:0009058 (biosynthetic process), GO:0016779 (nucleotidyltransferase activity)
*Aradu.8G9XJ*	1210713 to 1215639	Phosphopyruvate hydratase; IPR000941 (Enolase); GO:0000015 (phosphopyruvate hydratase complex), GO:0000287 (magnesium ion binding), GO:0004634 (phosphopyruvate hydratase activity), GO:0006096 (glycolysis)
*Aradu.2V08S*	1216357 to 1220881	Neutral ceramidase-like isoform 1 [Glycine max]; IPR006823 (Neutral/alkaline nonlysosomal ceramidase)
*Aradu.188J4*	1225601 to 1228083	ATP-dependent DNA helicase, putative *n* = 7 Tax = Trypanosomatidae RepID = E9BR42_LEIDB; IPR010339 (TIP49, C-terminal), IPR027238 (RuvB-like), IPR027417 (P-loop containing nucleoside triphosphate hydrolase); GO:0000166 (nucleotide binding), GO:0003678 (DNA helicase activity), GO:0005524 (ATP binding), GO:0017111 (nucleoside-triphosphatase activity), GO:0043141 (ATP-dependent 5’-3’ DNA helicase activity)
*Aradu.X1L7N*	1229461 to 1233773	Armadillo repeat-containing protein 8-like [Glycine max]; IPR016024 (Armadillo-type fold); GO:0005488 (binding), GO:0005515 (protein binding)
*Aradu.CH4M9*	1233738 to 1235758	Chaperonin-like RbcX protein; IPR003435 (Chaperonin-like RbcX)
*Aradu.8247R*	1242781 to 1253120	LRR and NB-ARC domain disease resistance protein; IPR000767 (Disease resistance protein), IPR013057 (Amino acid transporter, transmembrane), IPR025875 (Leucine rich repeat 4), IPR027417 (P-loop containing nucleoside triphosphate hydrolase); GO:0006952 (defense response), GO:0043531 (ADP binding)
*Aradu.1K902*	1254064 to 1256589	Transmembrane amino acid transporter family protein; IPR013057 (Amino acid transporter, transmembrane)
*Aradu.J21RS*	1258934 to 1263681	Transmembrane amino acid transporter family protein; IPR001854 (Ribosomal protein L29), IPR010729 (Ribosomal protein L47, mitochondrial), IPR013057 (Amino acid transporter, transmembrane); GO:0003735 (structural constituent of ribosome), GO:0005622 (intracellular), GO:0005761 (mitochondrial ribosome), GO:0005840 (ribosome), GO:0006412 (translation)
*Aradu.PWJ6Q*	1269328 to 1270490	Amino acid permease 7; IPR013057 (Amino acid transporter, transmembrane)
*Aradu.G4PX6*	1275878 to 1279624	Probable galactinol--sucrose galactosyltransferase 2-like isoform X1 [Glycine max]; IPR008811 (Glycosyl hydrolases 36), IPR013785 (Aldolase-type TIM barrel); GO:0003824 (catalytic activity)
*Aradu.WDU9W*	1283093 to 1284551	Class III homeobox-leucine zipper protein *n* = 2 Tax = Asparagus RepID = I0IUI4_9ASPA; IPR013978 (MEKHLA)
*Aradu.3FH81*	1292398 to 1292610	Transmembrane protein, putative
*Aradu.FX37I*	1294555 to 1304080	BTB/POZ domain-containing protein; IPR011333 (BTB/POZ fold); GO:0005515 (protein binding)
*Aradu.XJ0L1*	1305965 to 1306534	BTB/POZ domain-containing protein; IPR011333 (BTB/POZ fold); GO:0005515 (protein binding)
*Aradu.XLV7R*	1311374 to 1312674	Heavy metal transport/detoxification superfamily protein, putative *n* = 1 Tax = Theobroma cacao RepID = UPI00042B042A
*Aradu.JRV68*	1314901 to1315584	Late embryogenesis abundant (LEA) hydroxyproline-rich glycoprotein family; IPR004864 (Late embryogenesis abundant protein, LEA-14)

## Supplementary Materials

The following are available online at https://www.mdpi.com/2073-4425/11/12/1402/s1; Table S1. SSRs developed in this study; Table S2. Analysis of variance of pod and seed traits in NILs; Figure S1. Plots of validation of some SNPs; Supplementary File S1. Detailed explanation of the genotypic constitution of the line 1575-02 and the pattern of segregation in its offspring; Figure S2. Genetic map of the target region with the new SSR markers; Figure S3. Multiple mean comparison of pod and seed related traits between genotypes; Figure S4. Protein sequences alignment of the most promising candidate genes identified with their orthologues; Figure S5. Graphical representation of the genotype at the gene loci in two genes model with additive action.

## References

[1] Diamond J. (2002). Evolution, consequences and future of plant and animal domestication. Nat. Cell Biol..

[2] Doebley J.F., Gaut B.S., Smith B.D. (2006). The Molecular Genetics of Crop Domestication. Cell.

[3] Kochert G., Stalker H.T., Gimenes M., Galgaro L., Lopes C.R., Moore K. (1996). Rflp and Cytogenetic Evidence on the Origin and Evolution of Allotetraploid Domesticated Peanut, *Arachis hypogaea* (Leguminosae). Am. J. Bot..

[4] Grabiele M., Chalup L., Robledo G., Seijo G. (2012). Genetic and geographic origin of domesticated peanut as evidenced by 5S rDNA and chloroplast DNA sequences. Plant Syst. Evol..

[5] Bertioli D.J., Cannon S.B., Froenicke L., Huang G., Farmer A.D., Cannon E.K.S., Liu X., Gao D., Clevenger J., Dash S. (2016). The genome sequences of Arachis duranensis and Arachis ipaensis, the diploid ancestors of cultivated peanut. Nat. Genet..

[6] Bertioli D.J., Jenkins J., Clevenger J., Dudchenko O., Gao D., Seijo G., Leal-Bertioli S.C.M., Ren L., Farmer A.D., Pandey M.K. (2019). The genome sequence of segmental allotetraploid peanut *Arachis hypogaea*. Nat. Genet..

[7] Zhang C., Chen H., Yang M., Wang J., Pandey M.K., Zhang C., Chang W.-C., Zhang L., Zhang X., Tang R. (2019). The genome of cultivated peanut provides insight into legume karyotypes, polyploid evolution and crop domestication. Nat. Genet..

[8] Moretzsohn M.C., Gouvea E.G., Inglis P.W., Leal-Bertioli S.C.M., Valls J.F.M., Bertioli D.J. (2012). A study of the relationships of cultivated peanut (*Arachis hypogaea*) and its most closely related wild species using intron sequences and microsatellite markers. Ann. Bot..

[9] Yin D., Ji C., Song Q., Zhang W., Zhang X., Zhao K., Chen C.Y., Wang C., He G., Liang Z. (2019). Comparison of Arachis monticola with Diploid and Cultivated Tetraploid Genomes Reveals Asymmetric Subgenome Evolution and Improvement of Peanut. Adv. Sci..

[10] Leal-Bertioli S.C.M., Moretzsohn M.C., Santos S.P., Brasileiro A.C.M., Guimarães P.M., Bertioli D.J., Araujo A.C.G. (2017). Phenotypic effects of allotetraploidization of wild Arachis and their implications for peanut domestication. Am. J. Bot..

[11] Leal-Bertioli S.C.M., Bertioli D., Guimarães P.M., Pereira T.D., Galhardo I., Silva J.P., Brasileiro A.C.M., Oliveira R.S., Silva P.Í.T., Vadez V. (2012). The effect of tetraploidization of wild Arachis on leaf morphology and other drought-related traits. Environ. Exp. Bot..

[12] Fonceka D., Tossim H.-A., Rivallan R., Vignes H., Faye I., Ndoye O., Moretzsohn M.C., Bertioli D.J., Glaszmann J.-C., Courtois B. (2012). Fostered and left behind alleles in peanut: Interspecific QTL mapping reveals footprints of domestication and useful natural variation for breeding. BMC Plant Biol..

[13] Fonceka D., Tossim H.-A., Rivallan R., Vignes H., Lacut E., De Bellis F., Faye I., Ndoye O., Leal-Bertioli S.C.M., Valls J.F.M. (2012). Construction of Chromosome Segment Substitution Lines in Peanut (*Arachis hypogaea* L.) Using a Wild Synthetic and QTL Mapping for Plant Morphology. PLoS ONE.

[14] Tossim H.-A., Nguepjop J.R., Diatta C., Sambou A., Seye M., Sane D., Rami J.-F., Fonceka D. (2020). Assessment of 16 Peanut (*Arachis hypogaea* L.) CSSLs Derived from an Interspecific Cross for Yield and Yield Component Traits: QTL Validation. Agronomy.

[15] Shirasawa K., Koilkonda P., Aoki K., Hirakawa H., Tabata S., Watanabe M., Hasegawa M., Kiyoshima H., Suzuki S., Kuwata C. (2012). In silico polymorphism analysis for the development of simple sequence repeat and transposon markers and construction of linkage map in cultivated peanut. BMC Plant Biol..

[16] Chen W., Jiao Y., Cheng L., Huang L., Liao B., Tang M., Ren X., Zhou X., Chen Y., Jiang H. (2016). Quantitative trait locus analysis for pod- and kernel-related traits in the cultivated peanut (*Arachis hypogaea* L.). BMC Genet..

[17] Huang L., He H., Chen W., Ren X., Chen Y., Zhou X., Xia Y., Wang X., Jiang X., Liao B. (2015). Quantitative trait locus analysis of agronomic and quality-related traits in cultivated peanut (*Arachis hypogaea* L.). Theor. Appl. Genet..

[18] Chen Y., Ren X., Zheng Y., Zhou X., Huang L., Yan L., Jiao Y., Chen W., Huang S., Wan L. (2017). Genetic mapping of yield traits using RIL population derived from Fuchuan Dahuasheng and ICG6375 of peanut (*Arachis hypogaea* L.). Mol. Breed..

[19] Luo H., Ren X., Li Z., Xu Z., Li X., Huang L., Zhou X., Chen Y., Chen W., Lei Y. (2017). Co-localization of major quantitative trait loci for pod size and weight to a 3.7 cM interval on chromosome A05 in cultivated peanut (*Arachis hypogaea* L.). BMC Genom..

[20] Luo H., Liao B., Jiang H., Guo J., Ren X., Chen W., Huang L., Zhou X., Chen Y., Liu N. (2017). Chromosomes A07 and A05 associated with stable and major QTLs for pod weight and size in cultivated peanut (*Arachis hypogaea* L.). Theor. Appl. Genet..

[21] Wang Z., Huai D., Zhang Z., Cheng K., Kang Y., Wan L., Yan L., Jiang H., Lei Y., Liao B. (2018). Development of a High-Density Genetic Map Based on Specific Length Amplified Fragment Sequencing and Its Application in Quantitative Trait Loci Analysis for Yield-Related Traits in Cultivated Peanut. Front. Plant Sci..

[22] Khera P., Pandey M.K., Mallikarjuna N., Sriswathi M., Roorkiwal M., Janila P., Sharma S., Shilpa K., Sudini H., Guo B. (2018). Genetic imprints of domestication for disease resistance, oil quality, and yield component traits in groundnut (*Arachis hypogaea* L.). Mol. Genet. Genom..

[23] Zhang S., Hu X., Miao H., Chu Y., Cui F., Yang W., Wang C., Shen Y., Xu T., Zhao L. (2019). QTL identification for seed weight and size based on a high-density SLAF-seq genetic map in peanut (*Arachis hypogaea* L.). BMC Plant Biol..

[24] Chu Y., Chee P., Isleib T.G., Holbrook C.C., Ozias-Akins P. (2019). Major seed size QTL on chromosome A05 of peanut (*Arachis hypogaea*) is conserved in the US mini core germplasm collection. Mol. Breed..

[25] Gangurde S.S., Wang H., Yaduru S., Pandey M.K., Fountain J.C., Chu Y., Isleib T., Holbrook C.C., Xavier A., Culbreath A.K. (2019). Nested-association mapping (NAM)-based genetic dissection uncovers candidate genes for seed and pod weights in peanut (*Arachis hypogaea* L.). Plant Biotechnol. J..

[26] Chavarro C., Chu Y., Holbrook C., Isleib T., Bertioli D.J., Hovav R., Butts C., Lamb M., Sorensen R., Jackson S.A. (2020). Pod and Seed Trait QTL Identification To Assist Breeding for Peanut Market Preferences. G3.

[27] Jaganathan D., Bohra A., Thudi M., Varshney R.K. (2020). Fine mapping and gene cloning in the post-NGS era: Advances and prospects. Theor. Appl. Genet..

[28] Yang Q., Zhang N., Xu M.-L. (2012). A Sequential Quantitative Trait Locus Fine-Mapping Strategy Using Recombinant-Derived ProgenyF. J. Integr. Plant Biol..

[29] Cockram J., Mackay I. (2018). Genetic Mapping Populations for Conducting High-Resolution Trait Mapping in Plants. Adv. Biochem. Eng..

[30] Agarwal G., Clevenger J., Pandey M.K., Wang H., Shasidhar Y., Chu Y., Fountain J.C., Choudhary D., Culbreath A.K., Liu X. (2018). High-density genetic map using whole-genome resequencing for fine mapping and candidate gene discovery for disease resistance in peanut. Plant Biotechnol. J..

[31] Khan S.A., Chen H., Deng Y., Chen Y., Zhang C., Cai T., Ali N., Mamadou G., Xie D., Guo B. (2020). High-density SNP map facilitates fine mapping of QTLs and candidate genes discovery for Aspergillus flavus resistance in peanut (*Arachis hypogaea*). Theor. Appl. Genet..

[32] Fan C., Xing Y., Mao H., Lu T., Han B., Xu C., Li X., Zhang Q. (2006). GS3, a major QTL for grain length and weight and minor QTL for grain width and thickness in rice, encodes a putative transmembrane protein. Theor. Appl. Genet..

[33] Ding X., Li X., Xiong L. (2011). Evaluation of near-isogenic lines for drought resistance QTL and fine mapping of a locus affecting flag leaf width, spikelet number, and root volume in rice. Theor. Appl. Genet..

[34] Liu Y., Zhang A., Wang F., Kong D., Li M., Bi J., Zhang F., Wang J., Luo X., Pan Z. (2019). Fine mapping a quantitative trait locus, qSER-7, that controls stigma exsertion rate in rice (*Oryza sativa* L.). Rice.

[35] LeComte L., Saliba-Colombani V., Gautier A., Gomez-Jimenez M.C., Duffe P., Buret M., Causse M. (2004). Fine mapping of QTLs of chromosome 2 affecting the fruit architecture and composition of tomato. Mol. Breed..

[36] Djébali N., Jauneau A., Ameline-Torregrosa C., Chardon F., Jaulneau V., Mathé C., Bottin A., Cazaux M., Pilet-Nayel M.-L., Baranger A. (2009). Partial Resistance of Medicago truncatula to Aphanomyces euteiches Is Associated with Protection of the Root Stele and Is Controlled by a Major QTL Rich in Proteasome-Related Genes. Mol. Plant-Microbe Interact..

[37] Holbrook C.C., Timper P., Dong W., Kvien C.K., Culbreath A.K. (2008). Development of Near-Isogenic Peanut Lines with and without Resistance to the Peanut Root-Knot Nematode. Crop. Sci..

[38] Nagy E.D., Chu Y., Guo Y., Khanal S., Tang S., Li Y., Dong W.B., Timper P., Taylor C., Ozias-Akins P. (2010). Recombination is suppressed in an alien introgression in peanut harboring Rma, a dominant root-knot nematode resistance gene. Mol. Breed..

[39] Yeri S.B., Shirasawa K., Pandey M.K., Gowda M.V.C., Sujay V., Shriswathi M., Nadaf H.L., Motagi B.N., Lingaraju A.R.S., Bhat A.R.S. (2013). Development of NILs from heterogeneous inbred families for validating the rust resistance QTL in peanut (*Arachis hypogaea* L.). Plant Breed..

[40] Fonceka D., Tossim H.-A., Rivallan R., Faye I., Sall M.N., Ndoye O., Fávero A.P., Bertioli D.J., Glaszmann J.-C., Courtois B. (2009). Genetic mapping of wild introgressions into cultivated peanut: A way toward enlarging the genetic basis of a recent allotetraploid. BMC Plant Biol..

[41] Untergasser A., Cutcutache I., Koressaar T., Ye J., Faircloth B.C., Remm M., Rozen S.J. (2012). Primer3 - New capacities and interfaces. Nucleic Acids Res..

[42] Pandey M.K., Agarwal G., Kale S.M., Clevenger J., Nayak S.N., Sriswathi M., Chitikineni A., Chavarro C., Chen X., Upadhyaya H.D. (2017). Development and Evaluation of a High Density Genotyping ‘Axiom_Arachis’ Array with 58 K SNPs for Accelerating Genetics and Breeding in Groundnut. Sci. Rep..

[43] Lorieux M. (2012). MapDisto: Fast and efficient computation of genetic linkage maps. Mol. Breed..

[44] Voorrips R.E. (2002). MapChart: Software for the Graphical Presentation of Linkage Maps and QTLs. J. Hered..

[45] Leal-Bertioli S., Shirasawa K., Abernathy B., Moretzsohn M., Chavarro C., Clevenger J., Ozias-Akins P., Jackson S., Bertioli D. (2015). Tetrasomic Recombination Is Surprisingly Frequent in AllotetraploidArachis. Genetics.

[46] Nguepjop J.R., Tossim H.-A., Bell J.M., Rami J.-F., Sharma S., Courtois B., Mallikarjuna N., Sane D., Fonceka D. (2016). Evidence of Genomic Exchanges between Homeologous Chromosomes in a Cross of Peanut with Newly Synthetized Allotetraploid Hybrids. Front. Plant Sci..

[47] Xue W., Xing Y., Weng X., Zhao Y., Tang W., Wang L., Zhou H., Yu S., Xu C., Li X. (2008). Natural variation in Ghd7 is an important regulator of heading date and yield potential in rice. Nat. Genet..

[48] Fridman E., Liu Y.S., Carmel-Goren L., Gur A., Shoresh M., Pleban T., Eshed Y., Zamir D. (2002). Two tightly linked QTLs modify tomato sugar content via different physiological pathways. Mol. Genet. Genom..

[49] Li N., Li Y. (2014). Ubiquitin-mediated control of seed size in plants. Front. Plant Sci..

[50] Li N., Xu R., Li Y. (2019). Molecular Networks of Seed Size Control in Plants. Annu. Rev. Plant Biol..

[51] Xu L., Wei Y., Reboul J., Vaglio P., Shin T.-H., Vidal M., Elledge S.J., Harper J.W. (2003). BTB proteins are substrate-specific adaptors in an SCF-like modular ubiquitin ligase containing CUL-3. Nat. Cell Biol..

[52] Furukawa M., He Y.J., Borchers C.H., Xiong Y. (2003). Targeting of protein ubiquitination by BTB–Cullin 3–Roc1 ubiquitin ligases. Nat. Cell Biol..

[53] Tewari R., Bailes E., Bunting K.A., Coates J.C. (2010). Armadillo-repeat protein functions: Questions for little creatures. Trends Cell Biol..

[54] Strader L.C., Ritchie S., Soule J.D., McGinnis K.M., Steber C.M. (2004). Recessive-interfering mutations in the gibberellin signaling gene SLEEPY1 are rescued by overexpression of its homologue, SNEEZY. Proc. Natl. Acad. Sci. USA.

[55] Ariizumi T., Lawrence P.K., Steber C.M. (2011). The Role of Two F-Box Proteins, SLEEPY1 and SNEEZY, in Arabidopsis Gibberellin Signaling. Plant Physiol..

[56] Fuentes S., Ljung K., Sorefan K., Alvey E., Harberd N.P., Østergaard L. (2012). Fruit Growth in Arabidopsis Occurs via DELLA-Dependent and DELLA-Independent Gibberellin Responses. Plant Cell.

[57] Bandyopadhyay K., Ulucay O., Sakiroglu M., Udvardi M.K., Verdier J. (2016). Analysis of Large Seeds from Three Different Medicago truncatula Ecotypes Reveals a Potential Role of Hormonal Balance in Final Size Determination of Legume Grains. Int. J. Mol. Sci..

[58] Zhang Y., Ni Z., Yao Y., Nie X., Sun Q. (2007). Gibberellins and heterosis of plant height in wheat (*Triticum aestivum* L.). BMC Genet..

[59] Byzova M.V., Franken J., Aarts M.G., De Almeida-Engler J., Engler G., Mariani C., Campagne M.M.V.L., Angenent G. (1999). Arabidopsis STERILE APETALA, a multifunctional gene regulating inflorescence, flower, and ovule development. Genes Dev..

[60] Sicard A., Kappel C., Lee Y.W., Woźniak N.J., Marona C., Stinchcombe J.R., Wright S.I., Lenhard M. (2016). Standing genetic variation in a tissue-specific enhancer underlies selfing-syndrome evolution inCapsella. Proc. Natl. Acad. Sci. USA.

[61] Wang Z., Li N., Jiang S., Gonzalez N., Huang X., Wang Y., Inzé D., Li Y. (2016). SCFSAP controls organ size by targeting PPD proteins for degradation in Arabidopsis thaliana. Nat. Commun..

[62] Li N., Liu Z., Wang Z., Ru L., Gonzalez N., Baekelandt A., Pauwels L., Goossens A., Xu R., Zhu Z. (2018). STERILE APETALA modulates the stability of a repressor protein complex to control organ size in Arabidopsis thaliana. PLoS Genet..

[63] Yang L., Liu H., Zhao J., Pan Y., Cheng S., Lietzow C.D., Wen C., Zhang X., Weng Y. (2018). LITTLELEAF(LL) encodes a WD40 repeat domain-containing protein associated with organ size variation in cucumber. Plant J..

[64] Ge L., Yu J., Wang H., Luth D., Bai G., Wang K., Chen R. (2016). Increasing seed size and quality by manipulating BIG SEEDS1 in legume species. Proc. Natl. Acad. Sci. USA.

[65] Yin P., Ma Q., Wang H., Feng D., Wang X.-B., Pei Y., Wen J., Tadege M., Niu L., Lin H. (2020). SMALL LEAF AND BUSHY1 controls organ size and lateral branching by modulating the stability of BIG SEEDS1 in Medicago truncatula. New Phytol..

