# Supplementary material for: Fine-Mapping of a Wild Genomic Region Involved in Pod and Seed Size Reduction on Chromosome A07 in Peanut (Arachis hypogaea L.)
Source: Genes (Basel). 2020 Nov 25;11(12):1402. doi: 10.3390/genes11121402 (PMC7761091; doi:10.3390/genes11121402)
Supplement: Supplementary file 1 [file genes-11-01402-s001.zip › Supplementary File 1_Final.pdf]

**Supplementary File 1: Tetrasomic segregation pattern in the offspring of line 1575-02**

To further investigate the genotype of the line 1575-02 in the region delimited by the two SNPs Aradu_A07_1136308 and Aradu_A07_1148327, we analyzed the segregation patterns of these two markers in 20 offspring derived from the self-fertilization of the line.
The figure 1 shows the plot of signal intensities of the two SNPs. On these two plots, the progenies segregated in two classes, confirming that the line 1575-02 was not homozygous at these two markers. However, for both SNPs, the observed segregation ratio (Table A) did not follow the (¼ , ½, ¼) ratios expected in the case of the self-fertilization of an heterozygous genotype. Moreover, no offspring clustered with the 12CS_091 parental line.

**Table A: Observed phenotype frequency for SNPs Aradu_A07_1136308 and Aradu_A07_1148327**

| **Aradu_A07_1136308** | | **Aradu_A07_1148327** | |
|---|---|---|---|
| Phenotype | Observed | Phenotype | Observed |
| CCCC | 0 | AAAA | 0 |
| ACCC | 5 | GAAA | 5 |
| AACC | 15 | GGAA | 15 |
| AAAC | 0 | GGGA | 0 |
| AAAA | 0 | GGGG | 0 |

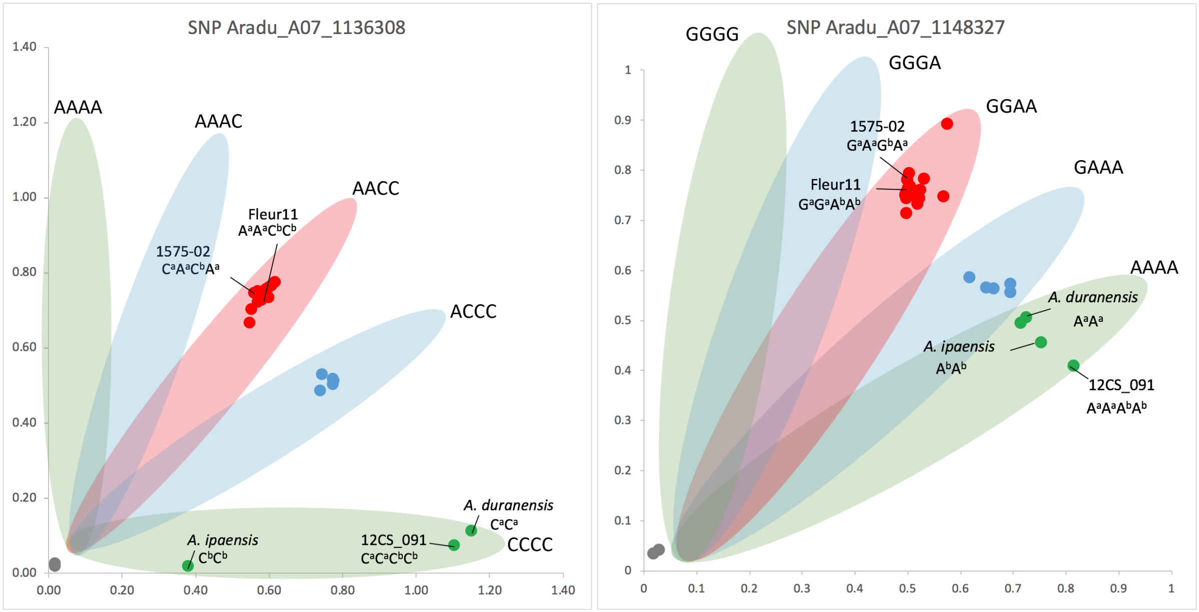


A B

Figure 1: Plots of signal intensities for SNPs Aradu_A07_1136308 (A) and Aradu_A07_1148327 (B) on 20 offspring derived from self-fertilization of line 1575-02. Fleur11, 12CS_091, 1575-02, A. duranensis and A. ipaensis are indicated on the plot with their expected genotypes. Expected or hypothesized tetraploid genotypes of these individuals are represented with the $A^aA^aC^bC^b$ notation. C/A and G/A represent the alleles at the SNP loci Aradu_A07_1136308 and Aradu_A07_1148327, respectively. The exponent letters a and b represent the peanut sub-genomes A and B, respectively. The light-green, light-blue and pink ellipses indicate the expected clusters (SNP phenotypes) in a tetrasomic segregation hypothesis.

Focusing on SNP Aradu_A07_1136308, since in the plot for analyzing the offspring 1575-02 clustered with Fleur 11, we hypothesized that the line had a $C^aA^aC^bA^a$ genotype arising from a tetrasomic recombination event. Starting from this genotype, we reconstructed the expected offspring's genotypes. The results are shown in the following tables. The Table B shows the expected genotypes under the assumption that all gametes are viable and can associate with one another. The frequency of each genotype was counted as well as the associated phenotype that can be observed in the SNP plot (Table C).

**Table B: Reconstruction of the genotypes that can be produced by line 1575-02 in a fully tetrasomic segregation**

| Gametes | $C^aC^b$ | $C^aA^a$ | $A^aC^b$ | $A^aA^a$ | $C^aA^a$ | $A^aC^b$ |
|---|---|---|---|---|---|---|
| $C^aC^b$ | $C^aC^aC^bC^b$ | $C^aC^aA^aC^b$ | $C^aA^aC^bC^b$ | $C^aA^aA^aC^b$ | $C^aC^aA^aC^b$ | $C^aA^aC^bC^b$ |
| $C^aA^a$ | $C^aC^aA^aC^b$ | $C^aC^aA^aA^a$ | $C^aA^aA^aC^b$ | $C^aA^aA^aA^a$ | $C^aC^aA^aA^a$ | $C^aA^aA^aC^b$ |
| $A^aC^b$ | $A^aC^aC^bC^b$ | $A^aC^aC^bA^a$ | $A^aA^aC^bC^b$ | $A^aA^aA^aC^b$ | $A^aC^aA^aC^b$ | $A^aA^aC^bC^b$ |
| $A^aA^a$ | $A^aC^aA^aC^b$ | $A^aC^aA^aA^a$ | $A^aA^aA^aC^b$ | $A^aA^aA^aA^a$ | $A^aC^aA^aA^a$ | $A^aA^aA^aC^b$ |
| $C^aA^a$ | $C^aC^aA^aC^b$ | $C^aC^aA^aA^a$ | $C^aA^aA^aC^b$ | $C^aA^aA^aA^a$ | $C^aC^aA^aA^a$ | $C^aA^aA^aC^b$ |
| $A^aC^b$ | $A^aC^aC^bC^b$ | $A^aC^aA^aC^b$ | $A^aA^aC^bC^b$ | $A^aA^aA^aC^b$ | $A^aC^aA^aC^b$ | $A^aA^aC^bC^b$ |

**Table C: Expected genotypes and phenotypes frequencies**

| **Genotypes** | **Frequency** | **Phenotypes** | **Frequency** |
|---|---|---|---|
| $C^aC^aC^bC^b$ | 1/36 | CCCC | 1/36 |
| $C^aC^aA^aC^b$ | 4/36 | ACCC | 8/36 |
| $C^aA^aC^bC^b$ | 4/36 | | |
| $C^aC^aA^aA^a$ | 4/36 | AACC | 18/36 |
| $A^aC^aA^aC^b$ | 10/36 | | |
| $A^aA^aC^bC^b$ | 4/36 | | |
| $A^aA^aA^aC^b$ | 4/36 | AAAC | 8/36 |
| $C^aA^aA^aA^a$ | 4/36 | | |
| $A^aA^aA^aA^a$ | 1/36 | AAAA | 1/36 |

In allotetraploid species, different genotypes at a given SNP marker can produce similar phenotypes. In this specific case, the nine possible genotypes correspond to 5 possible phenotypes in the SNP plot. Two phenotypes (CCCC and AAAA) are expected with a low frequency (1/36 each). They were not observed in the Aradu_A07_1136308 SNP plot (Figure A) probably because our sample size was reduced (20 offspring). The phenotypes CCCA and CAAA are expected with the same frequency (8/36), however, CAAA was not observed on the SNP plot while 5 offsprings had the CCCA phenotype. The absence of offspring in the CAAA cluster can be explained by the lethality of some gametic types or adverse selection in gametes association. To explore this hypothesis, we reconstructed the expected offspring's genotype assuming that the $A^aA^a$ gametes is unviable or adversely selected (Table D). The frequency of each genotype was counted as well as the associated phenotype that can be observed in the SNP plot (Table E).

**Table D: Reconstruction of the genotypes that can be produced by line 1575-02 in a tetrasomic segregation with lethality or adverse selection of gamete Aa**

| Gametes | $C^aC^b$ | $C^aA^a$ | $A^aC^b$ | $A^aA^a$ | $C^aA^a$ | $A^aC^b$ |
|---|---|---|---|---|---|---|
| $C^aC^b$ | $C^aC^aC^bC^b$ | $C^aC^aA^aC^b$ | $C^aA^aC^bC^b$ | - | $C^aC^aA^aC^b$ | $C^aA^aC^bC^b$ |
| $C^aA^a$ | $C^aC^aA^aC^b$ | $C^aC^aA^aA^a$ | $C^aA^aA^aC^b$ | - | $C^aC^aA^aA^a$ | $C^aA^aA^aC^b$ |
| $A^aC^b$ | $A^aC^aC^bC^b$ | $A^aC^aA^aC^b$ | $A^aA^aC^bC^b$ | - | $A^aC^aA^aC^b$ | $A^aA^aC^bC^b$ |
| $A^aA^a$ | - | - | - | - | - | - |
| $C^aA^a$ | $C^aC^aA^aC^b$ | $C^aC^aA^aA^a$ | $C^aA^aA^aC^b$ | - | $C^aC^aA^aA^a$ | $C^aA^aA^aC^b$ |
| $A^aC^b$ | $A^aC^aC^bC^b$ | $A^aC^aA^aC^b$ | $A^aA^aC^bC^b$ | - | $A^aC^aA^aC^b$ | $A^aA^aC^bC^b$ |

**Table E. Expected genotypes and phenotypes frequencies**

| Genotypes | Frequency | Phenotypes | Frequency |
|---|---|---|---|
| $C^aC^bC^aC^b$ | 1/25 | CCCC | 1/25 |
| $C^aC^aC^bA^a$ | 4/25 | ACCC | 8/25 |
| $C^aA^aC^bC^b$ | 4/25 | | |
| $C^aC^aA^aA^a$ | 4/25 | AACC | 16/25 |
| $C^aA^aA^aC^b$ | 8/25 | | |
| $A^aA^aC^bC^b$ | 4/25 | | |

Only 7 genotypic and 3 phenotypic classes are expected. These theoretical frequencies are very close to what we observed in the plot of signal intensity generated for SNP Aradu_A07_1136308 (Table F).

**Table F: Observed and expected phenotype frequency for SNP Aradu_A07_1136308**

| Phenotype | Observed | Expected |
|---|---|---|
| CCCC | 0 | 0.8 |
| ACCC | 5 | 6.4 |
| AACC | 15 | 12.8 |

The same rationale and conclusions apply to the Aradu_A07_1148327 SNP.

**Conclusion**

The line 1575_02 derived from a tetrasomic recombination between A and B genomes. It has AABA genomic composition at between the SNPs Aradu_A07_1136308 and Aradu_A07_1148327. The segregation pattern in the offspring is probably associated with gametes lethality or adverse selection.
