# Supplementary figures and images for: Fine-Mapping of a Wild Genomic Region Involved in Pod and Seed Size Reduction on Chromosome A07 in Peanut (Arachis hypogaea L.)
Source: Genes (Basel). 2020 Nov 25;11(12):1402. doi: 10.3390/genes11121402 (PMC7761091; doi:10.3390/genes11121402)

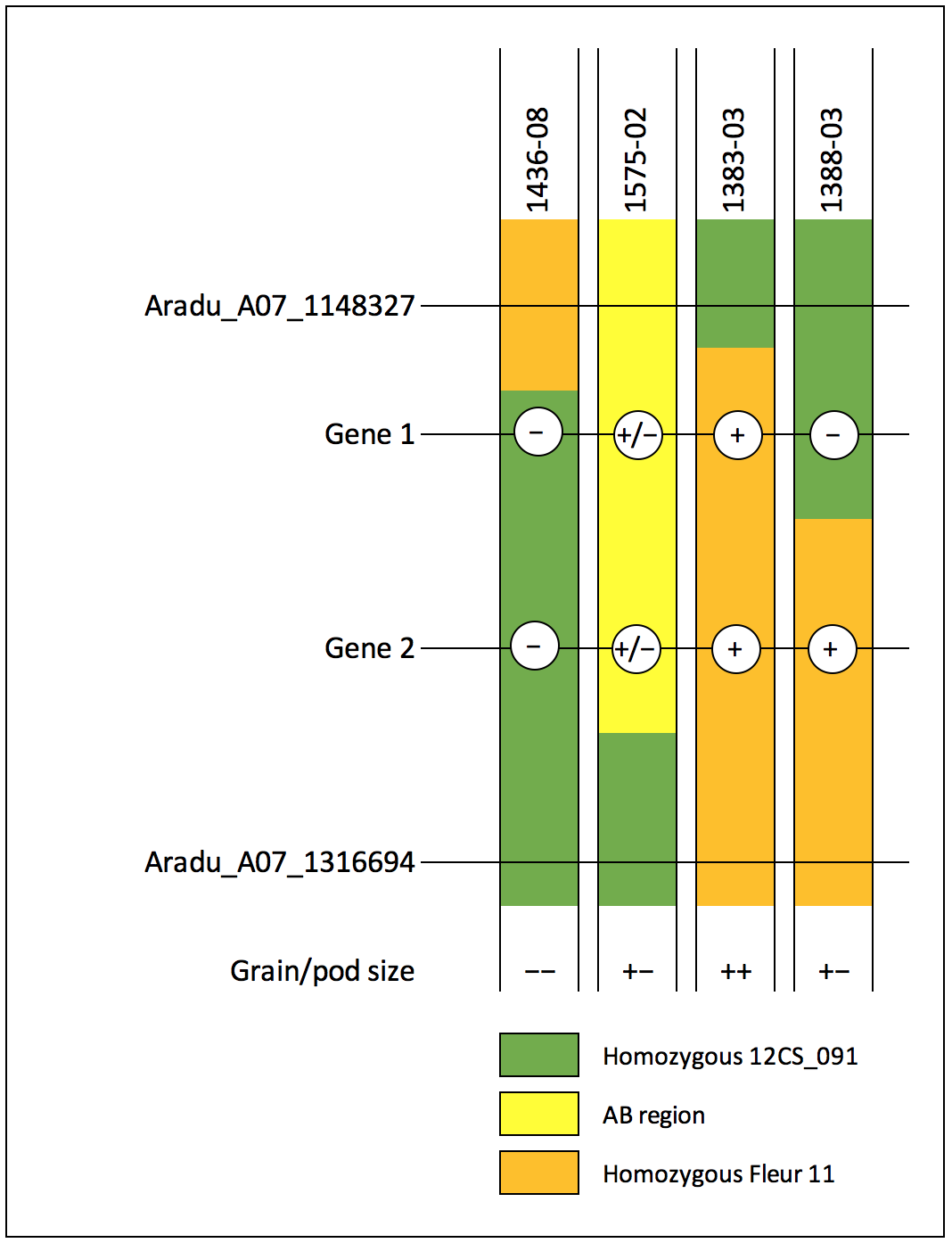

Supplement: Supplementary file 1 [file genes-11-01402-s001.zip › figureS5-new.png]

**cM****Loci**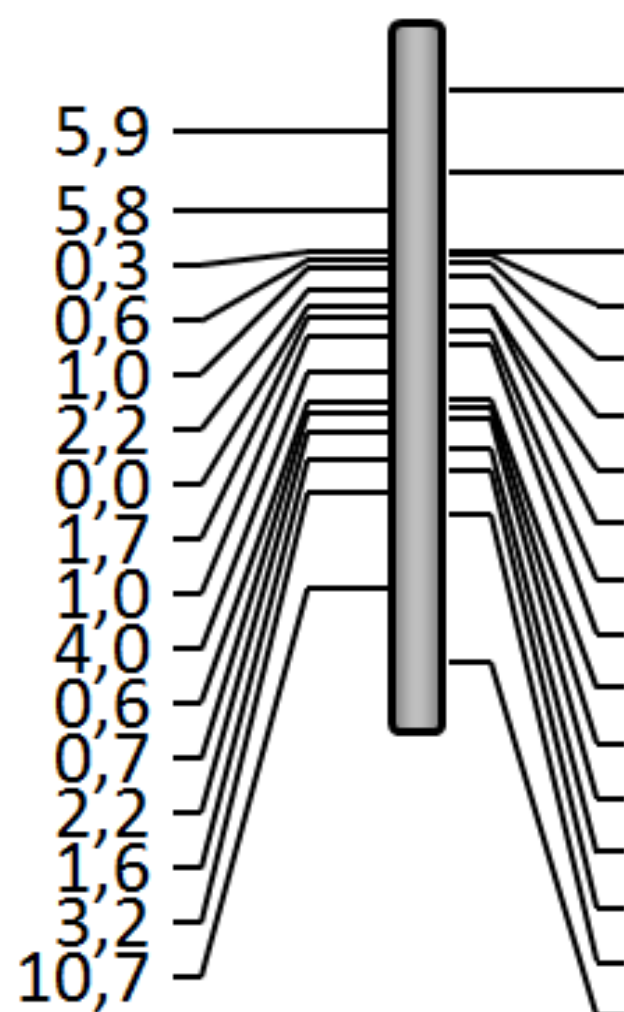

(1) RN13D04 \*\*\*\*\*  
(2) Seq2E06 \*\*  
(5) CS91\_A7-06 \*\*\*\*\*  
(3) CS91\_A7-04 \*\*\*  
(16) CS91\_A7-25 \*\*\*  
(15) CS91\_A7-23 \*\*  
(13) CS91\_A7-20 \*\*\*  
(9) CS91\_A7-13 \*\*\*  
(10) CS91\_A7-14 \*\*  
(14) CS91\_A7-21 \*\*  
(4) CS91\_A7-05 \*  
(6) CS91\_A7-07 \*  
(8) CS91\_A7-10  
(11) CS91\_A7-17  
(12) CS91\_A7-18  
(7) CS91\_A7-08  
(17) CS91\_A7-30 \*\*\*\*\*

Supplement: Supplementary file 1 [file genes-11-01402-s001.zip › figureS2.pdf]

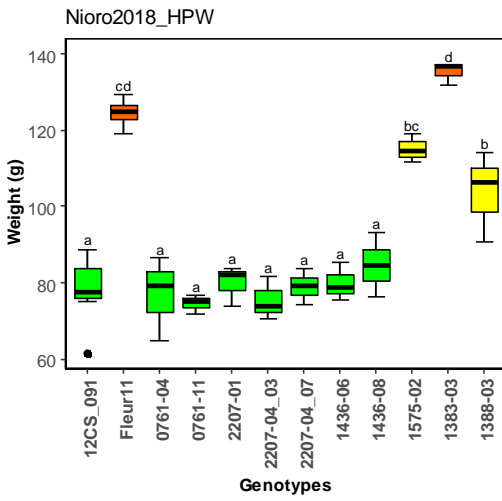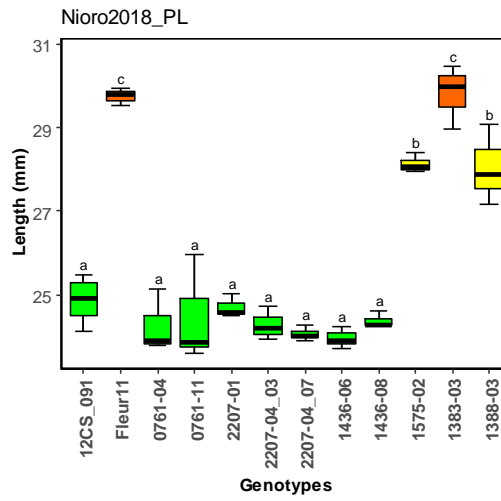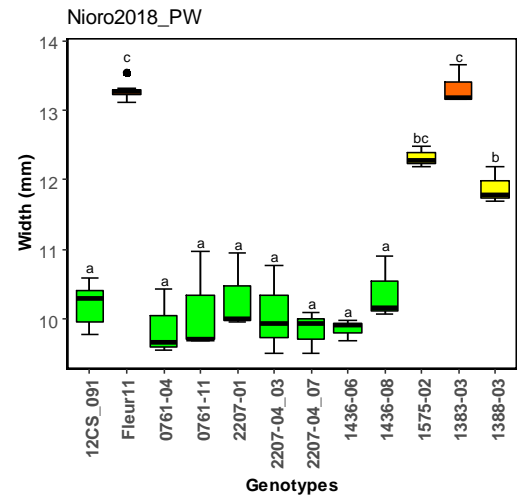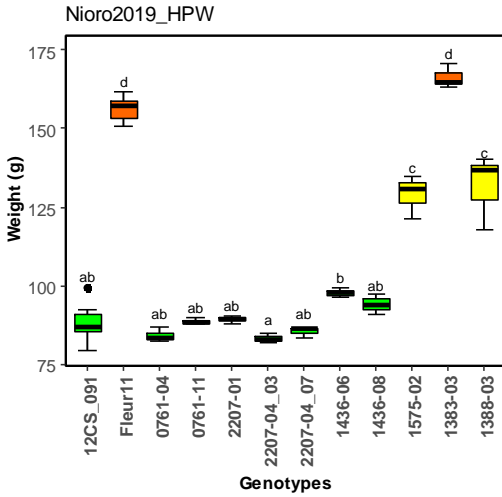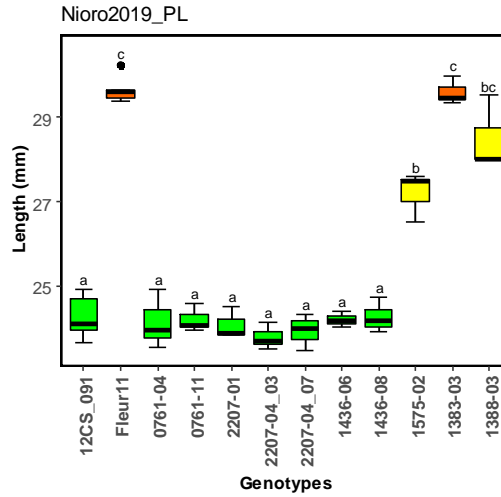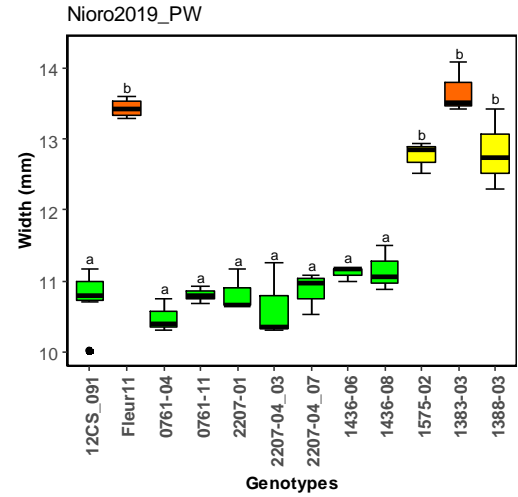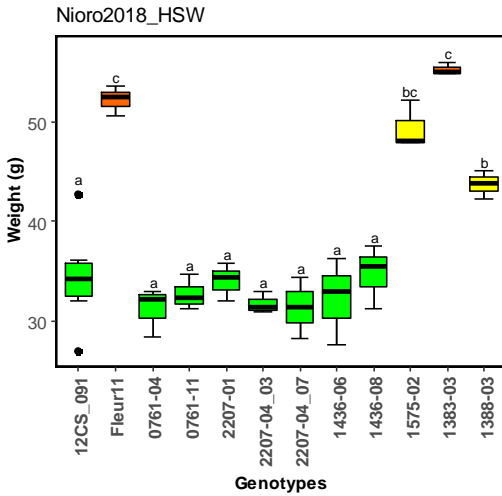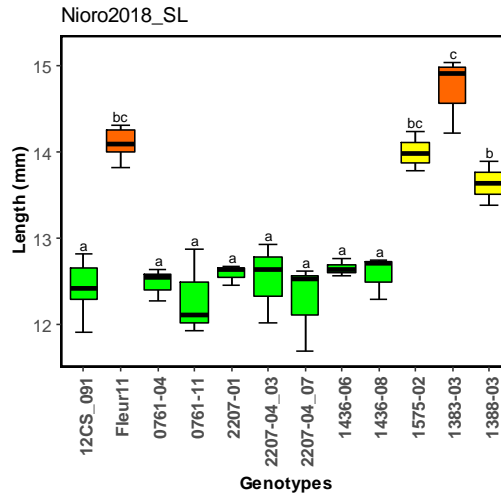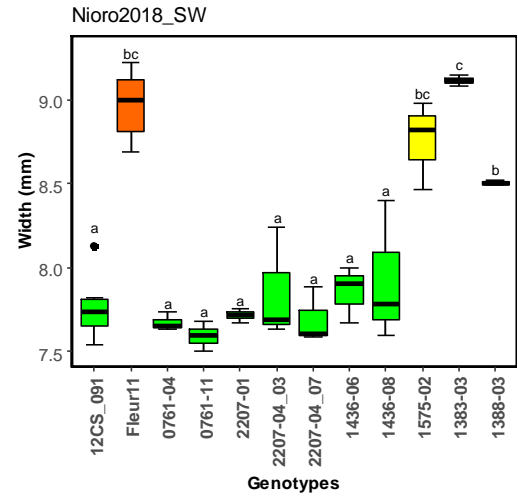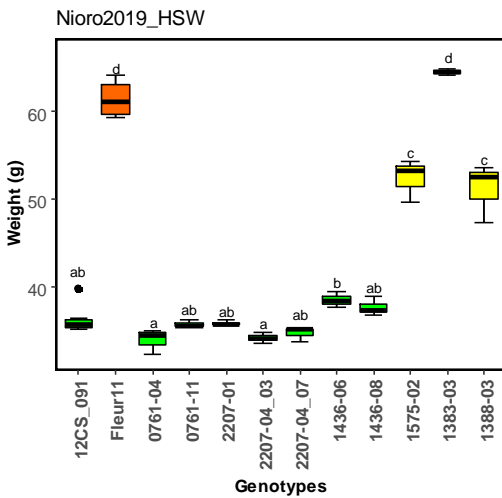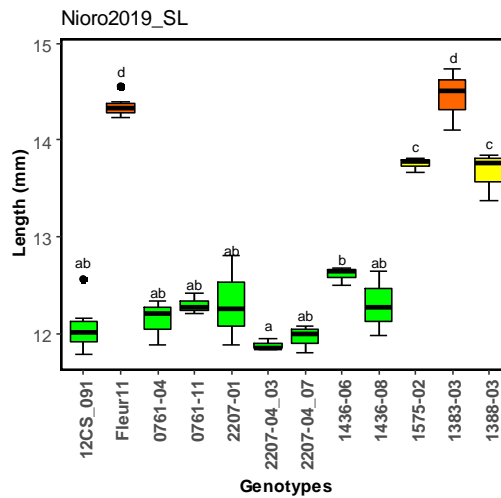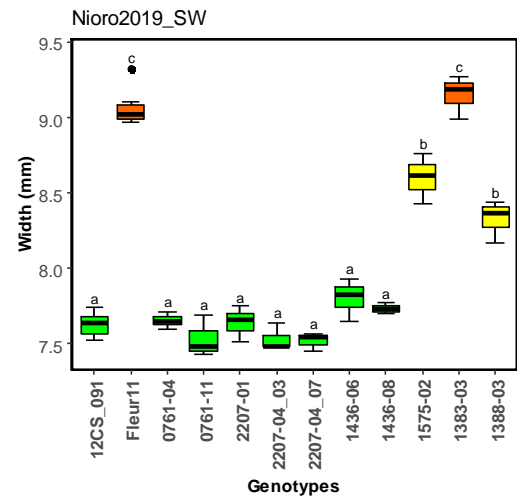

Supplement: Supplementary file 1 [file genes-11-01402-s001.zip › figureS3.pdf]

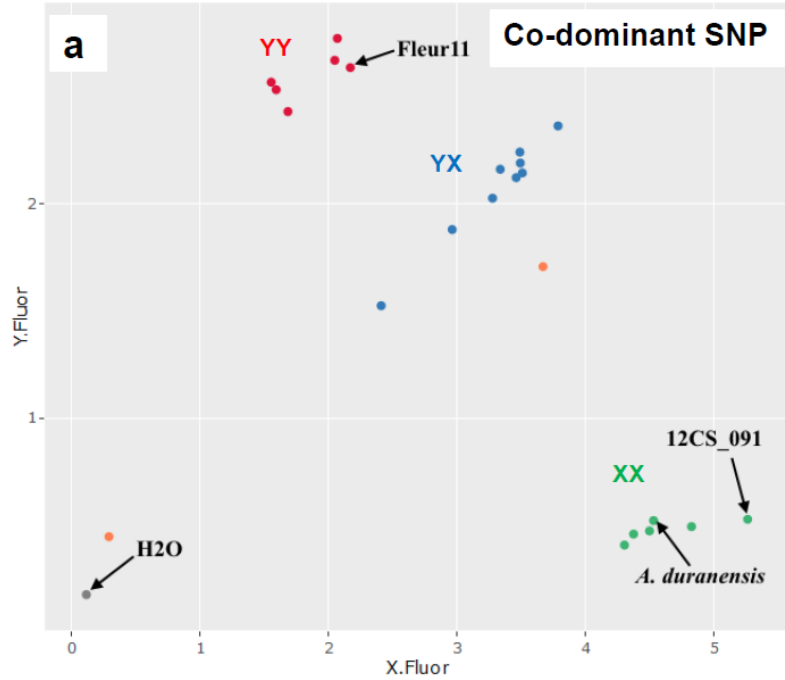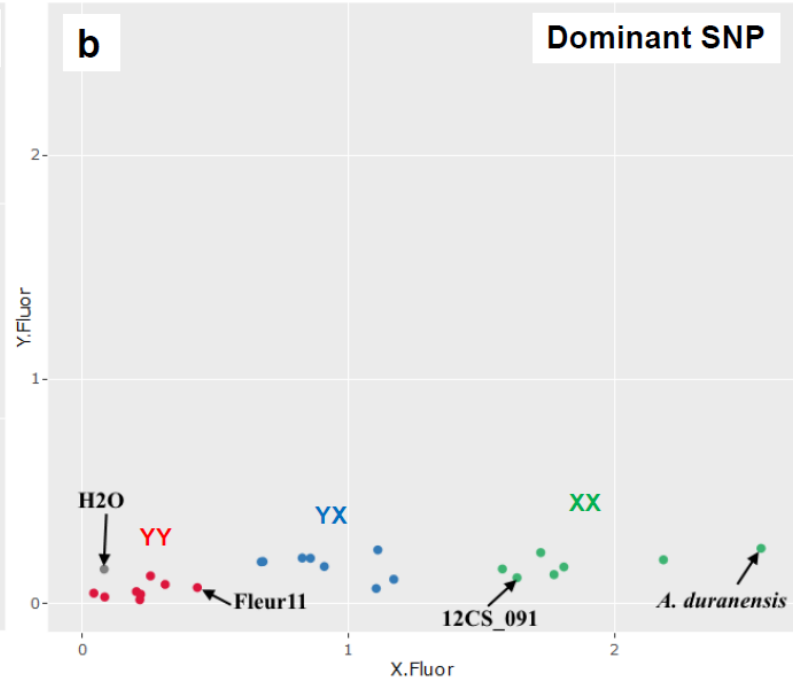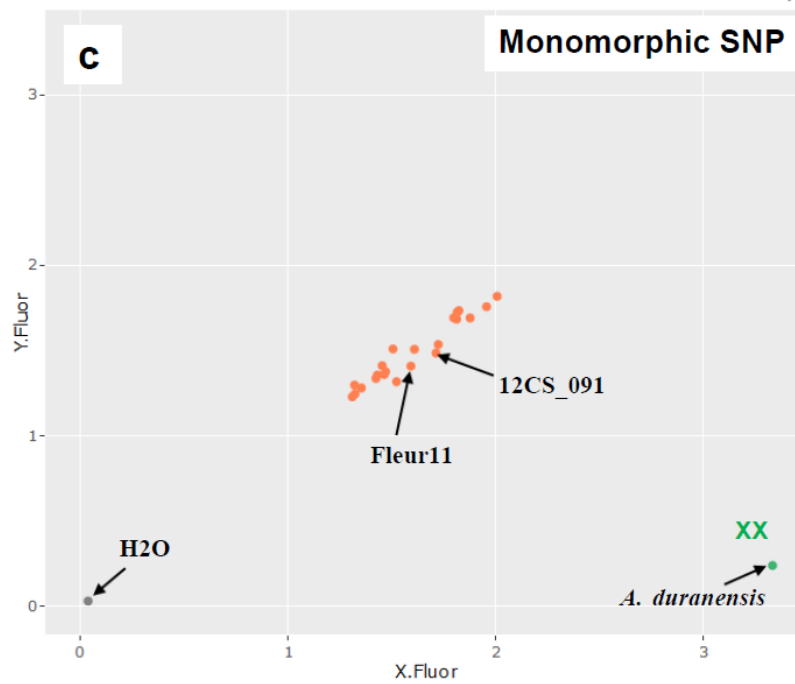

Supplement: Supplementary file 1 [file genes-11-01402-s001.zip › figureS1.pdf]

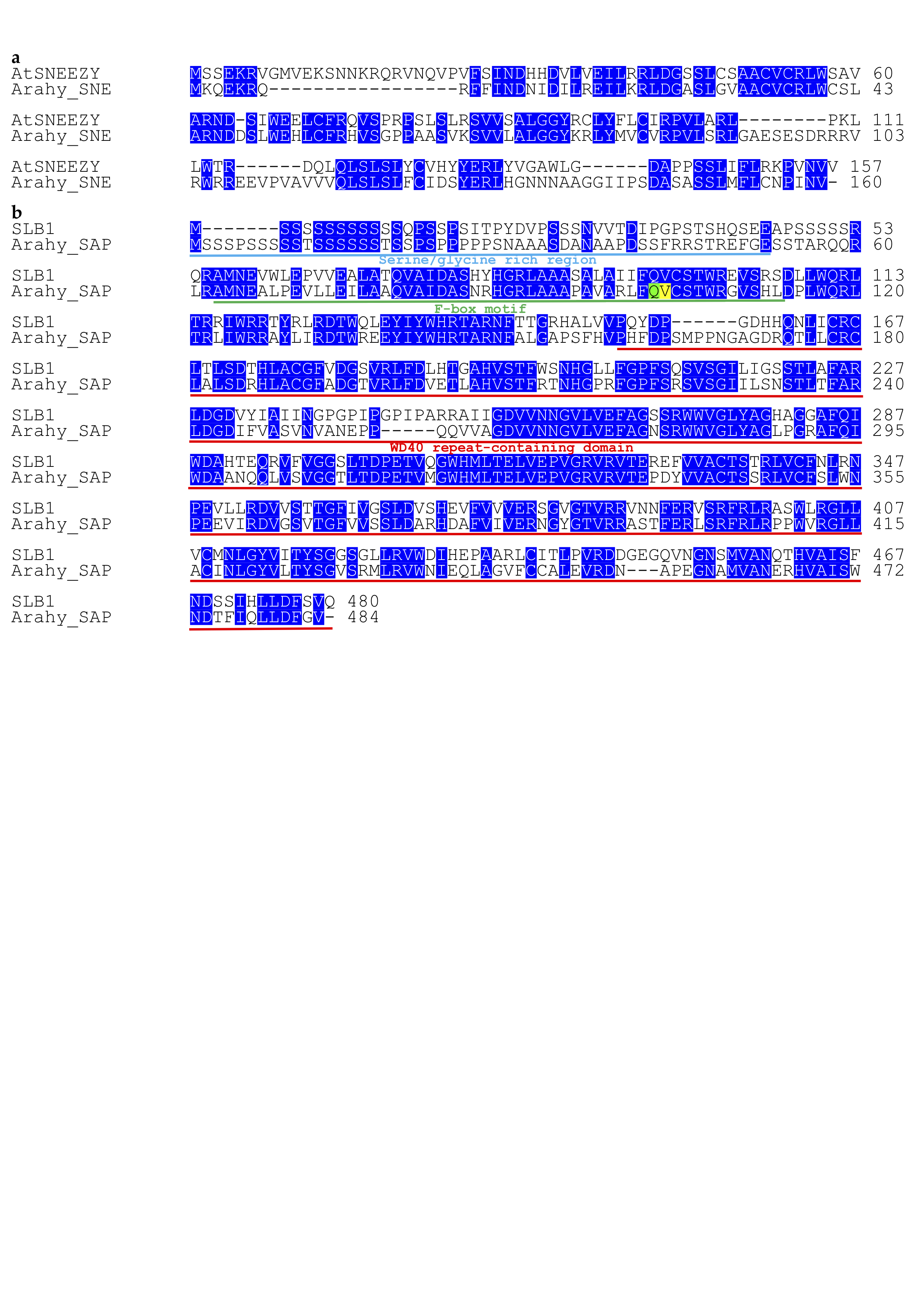

Supplement: Supplementary file 1 [file genes-11-01402-s001.zip › figureS4.png]
